# Leucine rich alpha-2-glycoprotein 1 (Lrg1) silencing protects against sepsis-mediated brain injury by inhibiting transforming growth factor beta1 (TGFβ1)/SMAD signaling pathway

**DOI:** 10.1080/21655979.2022.2048775

**Published:** 2022-03-10

**Authors:** Youhan Miao, Meihua Wang, Xiaojuan Cai, Qiqi Zhu, Liping Mao

**Affiliations:** Department of Infectious Diseases, The Third People’s Hospital of Nantong, Nantong, Jiangsu, China

**Keywords:** Sepsis-associated encephalopathy, lrg1, behavioral change, apoptosis, inflammatory response, TGFβ1/SMAD signaling

## Abstract

Sepsis-associated encephalopathy (SAE) is key manifestation of sepsis which is responsible for increased morbidity and mortality. Leucine rich alpha-2-glycoprotein 1 (Lrg1) is a secreted protein implicated in a variety of diseases. We aimed to explore the effects and potential mechanism of Lrg1 on sepsis-mediated brain injury. A sepsis-induced brain damage mice model was established. Then, ELISA was utilized to detect the levels of inflammatory factors in brain tissues. Behavioral performance, spatial learning and memory of mice were evaluated by open field test and Morris water maze test. The number of neurons was tested by H&E staining. Lrg1 expression was evaluated by RT-qPCR and western blot. In vitro, mouse hippocampal neuronal cell line (HT22) was stimulated by lipopolysaccharide (LPS). After Lrg1 silencing, cell viability was determined using CCK-8 and cell apoptosis was assessed by TUNEL. The levels of inflammatory factors were detected by ELISA. Moreover, western blot was applied to analyze the expression of proteins in transforming growth factor beta1 (TGFβ1)/SMAD signaling. Results revealed that mice in the model group showed obvious behavioral changes. Lrg1 was highly expressed in the brain tissues of model mice. Besides, Lrg1 knockdown suppressed the inflammation and apoptosis of LPS-induced HT22 cells. Moreover, Lrg1 silencing caused the inactivation of TGFβ1/SMAD signaling. Rescue assays confirmed that TGFβ1 overexpression reversed the impacts of Lrg1 deletion on the inflammation and apoptosis in LPS-induced HT22 cells. Collectively, Lrg1 silencing alleviates brain injury in SAE via inhibiting TGFβ1/SMAD signaling, implying that Lrg1 might serve as a promising target for SAE treatment.

## Graphical abstract

**Figure:**
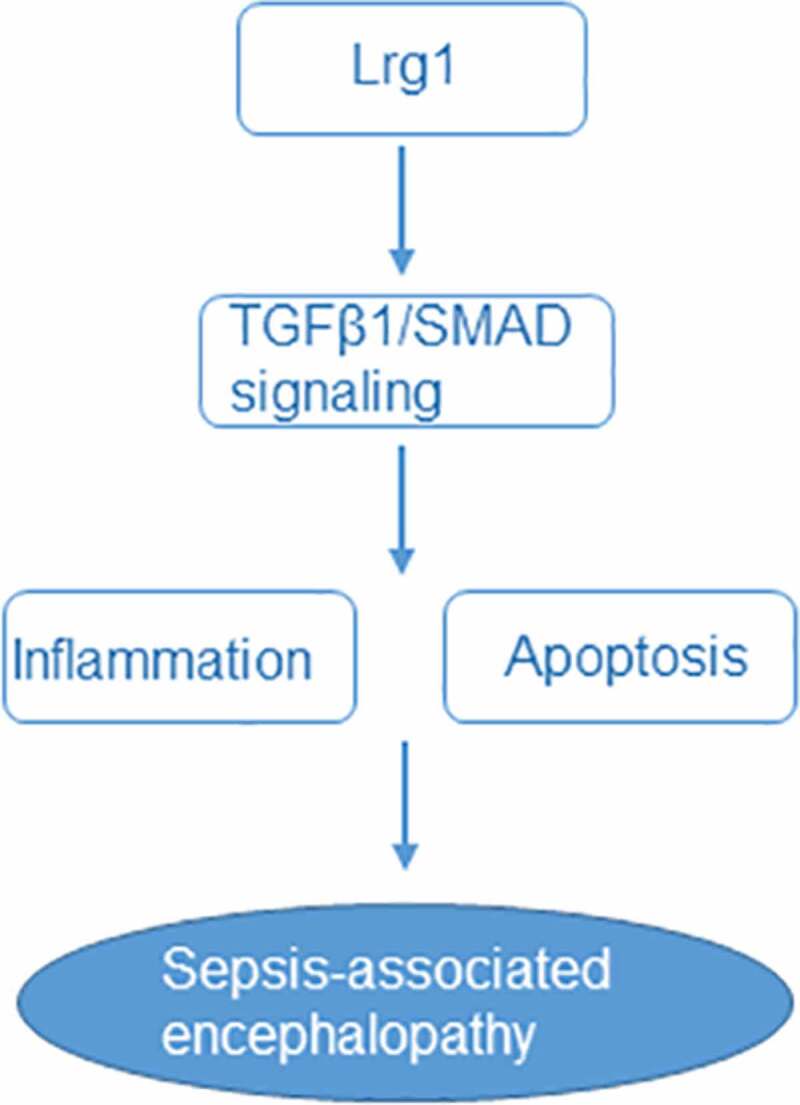

## Introduction

Sepsis, a serious medical condition that is frequently diagnosed in intensive care units (ICUs) patients, is recognized as a global health priority by the World Health Organization [1]. It is considered that sepsis poses a growing economic burden on health care systems in that patients with sepsis are prone to develop complications, have a higher mortality rate and face higher healthcare costs and longer treatment [2,3]. As a severe manifestation of sepsis, sepsis-associated encephalopathy (SAE) is characterized by a diffuse cerebral dysfunction caused by the systemic response to the infection without direct central nervous system (CNS) infection [4]. It is also associated with increased mortality, long-term cognitive disorder, and focal neurological deficits [5]. The most typical clinical feature of SAE is an altered mental status which ranges from mildly reduced awareness to convulsions, even deep coma [6]. Multiple factors including neurotransmitter dysfunction, inflammatory and ischemic lesions to the brain, microglial activation, and blood-brain barrier dysfunction have been reported to contribute to SAE, thus making the pathophysiology of SAE more complex [7]. Hence, it is necessary to investigate possible molecular markers for therapeutic methods from a novel perspective to improve the diagnosis and therapy of SAE.

Leucine rich alpha-2-glycoprotein 1 (Lrg1) is a secreted glycoprotein which was firstly isolated from human serum in 1977 [8]. It belongs to the leucine-rich repeat protein family and participates in various cellular processes including inflammation, angiogenesis, allergic responses [^9–12^]. Feng et al. have manifested that Lrg1 suppresses the viability but induces the apoptosis and autophagy of H9c2 cells under hypoxia conditions [13]. Meanwhile, patients who suffer from sepsis have increased serum levels of Lrg1 [14]. More importantly, Lrg1 has been identified as a novel potential biomarker for sepsis through bioinformatics analysis [15]. In line with this, Lrg1 was also discovered to be highly expressed in the brain tissues of SAE mice through GEO2R analysis. Additionally, compelling evidence indicate that Lrg1 can promote apoptosis and autophagy to exacerbate ischemia/reperfusion-induced brain injury through the transforming growth factor beta (TGFβ)/SMAD signaling pathway [16]. Thereafter, we infer that Lrg1 may play an important role in the regulation of SAE.

The present study aimed to explore whether Lrg1 can regulate SAE *in vivo* and *in vitro* as well as determine its potential regulatory mechanism related to TGFβ1/SMAD signaling. Our findings might provide a promising target for SAE treatment.

## Materials and methods

### Construction of sepsis model in mice

A total of 20 male C57BL/6 J mice (5–6 weeks old, 18–20 g) provided by Shanghai SLAC Laboratory Animal Company Limited (Shanghai, China) were housed under the condition of 21–23°C and a humidity of 55–60% with a 12-h light-dark cycle and free access to food and water. Then, animals were anesthetized by intraperitoneal injection of 35 mg/kg pentobarbital sodium, followed which the abdominal area of sepsis model mice was shaved and sterilized. Then, cecum was exposed for 15 min and ligated at 2/3 and punctured once with a 27-gauge needle [17]. The wound was then disinfected and closed utilizing a simple suture. The same surgical procedure was operated on Sham group mice without the ligation or perforation of the cecum. 48 h after CLP surgery, mice in each group were subjected to behavioral performance. Then, the brain tissues were collected to perform other experiments for further detection. Animal study was approved by the Medical Ethics Committee of The Third People’s Hospital of Nantong.

## Open field test (OFT)

The test was carried out in an open-field apparatus consisting of a 30 cm×30 cm wooden square surrounded by a dark wall with a hight of 30 cm. At the beginning, the mice were placed in the center of the open field. During the test, the mice were allowed to move freely around the open field within 5 min [17]. Their locomotor activity was recorded by a video camera (LS903, LG Electronics, Daejeon, Korea). The video tracking program EthoVision XT5 (Noldus Information Technology, Wageningen, NL, Canada) was used to measure the total distance traveled.

## Morris water maze (MWM)

Cognitive impairment in mice was evaluated by MWM assay. In brief, the water maze was consisted of a round, painted pool filled with water and maintained at 25°C. At the beginning of the experiment, mice were trained for 4 days to find the platform. After that, skimmed milk powder was added to the water. The time at which mice found the platform was recorded, and if it exceeded 60s, it was recorded as 60s [18]. The performance was conducted five times a day for 5 consecutive days. The escape latency of each mouse was recorded and analyzed by VideoMot software version 2.4.50923 (TSE Systems GmbH, Bad Homburg, Germany).

## Hematoxylin and Eosin (H&E) staining

H&E analysis was to assess pathological changes in the neurons of hippocampal tissues using a light microscopy (Olympus Corporation). The hippocampal tissues were fixed with 4% formaldehyde, then dehydrated with a series of different concentrations of ethanol, followed by being embedded into paraffin and then cut into 4 μm sections. Then hematoxylin solution (H8070; Solarbio) and eosin (Solarbio Life Sciences) were applied to stain the slices.

## Cell culture

Mouse hippocampal neuronal cell line (HT22) was purchased from Wuhan Hycell biotech and maintained in Dulbecco’s modified Eagle’s medium (DMEM; Gibco, Grand Island, NY, USA) added with 10% fetal bovine serum (FBS; Gibco, Grand Island, NY, USA) and 1% penicillin/streptomycin (Beyotime Institute of Biotechnology, China) at 37°C in a humid incubator with 5% CO_2_. To construct sepsis model in vitro, 1 μg/mL lipopolysaccharide (LPS; Sigma-Aldrich, St. Louis, MO, USA) was used to treat HT22 cells for 24 h [19,20].

## Plasmid transfection

Small interfering (si) RNAs targeting Lrg1 (si-Lrg1-1 and si-Lrg1-2) and the corresponding negative control (si-NC), TGFβ1 overexpression plasmid (Oe-TGFβ1) and the empty vector plasmid (Oe-NC) were obtained from Shanghai GenePharma Co., Ltd (Shanghai, China) [21]. Plasmid transfection was conducted with the employment of Lipofectamine® 3000 (Invitrogen, Carlsbad, CA, USA) following the standard manufacturer’s protocol. The transfection efficiency was evaluated by reverse transcription-quantitative polymerase chain reaction (RT-qPCR).

## Cell Counting Kit-8 (CCK-8) assay

Transfected HT22 cells were seeded into 96-well plates at a density of 2 × 10^4^ cells/well and cultured at 37°C overnight. Then, 10 μl CCK-8 solution (Yeasen Biotechnology Co., Shanghai, China) was added to each well. After an additional incubation at 37°C for 2 h, the cell viability at the absorbance of 450 nm was detected using a microplate reader (Bio-Rad, Hercules, CA, USA).

## Terminal-deoxynucleoitidyl transferase mediated nick end labeling (TUNEL) staining

Briefly, cells were washed with phosphate buffer solution (PBS) for three times and fixed with 4% paraformaldehyde, followed by the treatment with 0.5% Triton X-100. Cells were then incubated with 50 μl TUNEL reaction mixture (Roche, Basel, Switzerland) for 1 h at 37°C and nuclear staining was performed with 4’,6-diamidino-2-phenylindole (DAPI). The TUNEL-positive cells were observed under a fluorescence microscope (Olympus Corporation, Tokyo, Japan).

## Enzyme-Linked Immunosorbent Assay (ELISA)

Levels of inflammatory factors, including tumor necrosis factor-α (TNF-α), interleukin-6 (IL-6), interleukin-1β (IL-1β) and Interleukin 18 (IL-18), in hippocampal tissues of mice in each group and culture medium of HT22 cells were assessed with ELISA kits purchased from Shanghai XiTang Biotechnology (Shanghai, China) according to the standard protocol. The optical density values at 450 nm were read on a plate reader.

## RT-qPCR

After the isolation of total RNA from hippocampal tissues or HT22 cells with the aid of TRIzol Reagent (Invitrogen, Carlsbad, CA, USA), total RNA was reversely transcribed to complementary DNA (cDNA) by the PrimeScriptTM RT Reagent Kit (TaKaRa, Tokyo, Japan). PCR was performed using the SYBR ExScript qRT-PCR Kit (TaKaRa, Tokyo, Japan) on an Applied Biosystems (ABI) PCR System 7500 (ABI; Foster City, CA, USA). The PCR procedures were as follows: denaturation at 94°C for 2 min, amplification at 94°C for 30 sec (30 cycles), annealing at 58°C for 30 sec and extension at 72°C for 1 min, terminal elongation at 72°C for 10 min. The primer sequences used in this study were as follow: Lrg1, forward 5’-TCCACTCGCCACAACTCTTC-3’ and reverse 5’-GTCAGCCTAGGAGCCGTTTT-3’; Glyceraldehyde-3-phosphate dehydrogenase (GAPDH), forward 5’-CTACCCCCAATGTGTCCGTC-3’ and reverse 5’-GGCCTCTCTTGCTCAGTGTC-3’. Relative gene expression was figured out on the basis of 2^−ΔΔCt^ method [22]. GAPDH was designated as the internal control.

## Western blot

The total proteins were collected from hippocampal tissues or cell extracts by radioimmunoprecipitation (RIPA) lysis buffer (Beyotime Institute of Biotechnology, China). After being loaded on 10% sodium dodecyl sulfate-polyacrylamide gel electrophoresis (SDS-PAGE), protein samples were transferred onto polyvinylidene fluoride (PVDF) membranes (Millipore, Bedford, MA). The membranes were incubated with primary antibodies against Lrg1 (Abcam, Cambridge, UK; 1:1000, ab178698), B cell lymphoma-2 (Bcl-2) (Abcam, Cambridge, UK; 1:1000, ab32124), BCL-2 associated X (Bax) (Abcam, Cambridge, UK; 1:1000, ab32503), cleaved caspase 3 (Abcam, Cambridge, UK; 1:500, ab32042), caspase 3 (Abcam, Cambridge, UK; 1:5000, ab32351), TGFβ1 (Abcam, Cambridge, UK; 1:1000, ab215715), phosphorylated SMAD family member 2 (p-SMAD2) (Abcam, Cambridge, UK; 1:1000, ab280888), SMAD2 (Abcam, Cambridge, UK; 1:2000, ab40855), phosphorylated SMAD family member 3 (p-SMAD3) (Abcam, Cambridge, UK; 1:2000, ab52903), SMAD3 (Abcam, Cambridge, UK; 1:1000, ab40854) and GAPDH (Abcam, Cambridge, UK; 1:1000, ab8245) at 4°C overnight after being blocked with 5% nonfat milk. On the next day, the membranes were incubated with HRP-conjugated secondary antibodies (Abcam, Cambridge, UK; 1:2000, ab6789). The protein bands were visualized by the enhanced chemiluminescence (ECL; Millipore, USA). GAPDH was used as the internal control.

## Statistical analysis

All data was calculated using GraphPad Prism 8.0 software (GraphPad Software, San Diego, CA, USA) and presented as the mean ± standard deviation (SD). All experiments were respectively repeated in triplicate. Student’s t-test was employed to compare differences between two groups while differences among multiple groups were compared via one-way analysis of variance (ANOVA) with a Tukey’s post-hoc test. P < 0.05 was considered to indicate a statistically significant difference.

## Results

### Sepsis model mice exhibits brain behavioral changes

**Figure 1.**
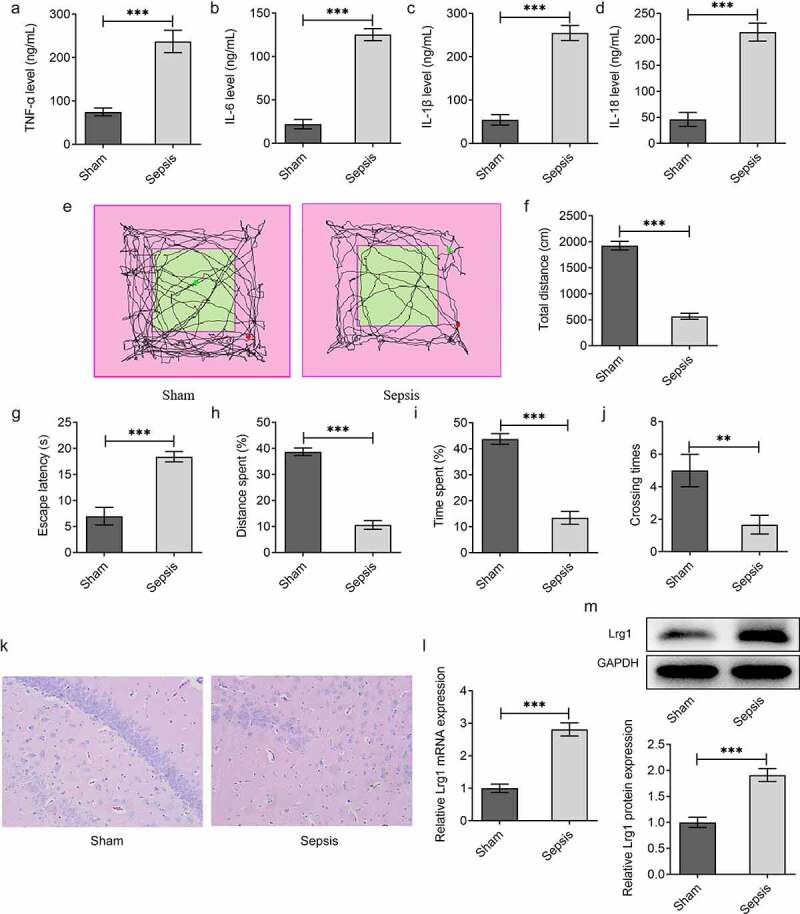
Sepsis model mice exhibit brain behavioral changes. The levels of inflammatory factors (a) TNF-α, (b) IL-6, (c) IL-1β and (d) IL-18 were assessed by ELISA analysis. (E-F) OFT was to detect the spontaneous viability of sepsis mice. (G-J) MWM was to explore the cognitive function of sepsis mice. (k) H&E staining was to evaluate the number of neurons of hippocampal tissues in sepsis mice. (Magnification, x200). (l) RT-qPCR and (m) western blot were respectively to analyze Lrg1 mRNA and protein expression. **P<0.01, ***P<0.001.

To investigate the role of Lrg1 in SAE, the *in vivo* mouse model of sepsis was firstly established. ELISA analysis was employed to test the levels of inflammatory factors including TNF-α, IL-6, IL-1β and IL-18. The results uncovered that the levels of TNF-α, IL-6, IL-1β and IL-18 were all elevated in the Sepsis group in comparison with the Sham group (Figure 1(a-d)). Moreover, OFT demonstrated that the total distance in the Sepsis group was significantly reduced compared with the Sham group, indicating that the spontaneous exploration of mice in the Sepsis group was observably suppressed in contrast with that in the Sham group (Figure 1(e-f)). Similarly, through MWM assay, it was observed that the escape latency, distance spent, time spent and crossing times of sepsis model mice were all greatly declined in comparison with the Sham group, which indicated that the cognitive function of sepsis mice was prominently decreased (Figure 1(G-J)). Moreover, as displayed in Figure 1(k), the number of neurons of hippocampal tissues in the Sepsis group was smaller than that in the Sham group. Besides, Lrg1 expression in sepsis mice brain tissues was examined by RT-qPCR and western blot analysis and the results manifested that Lrg1 was distinctly up-regulated in brain tissues of sepsis mice (Figure 1(l-m)). Overall, above results indicated that sepsis might lead to inflammatory response, cognitive dysfunction and neuron injury as well as limited spontaneous exploration.

## Silencing of Lrg1 ameliorates the inflammation and apoptosis of LPS-induced mouse hippocampal neuronal cells

**Figure 2.**
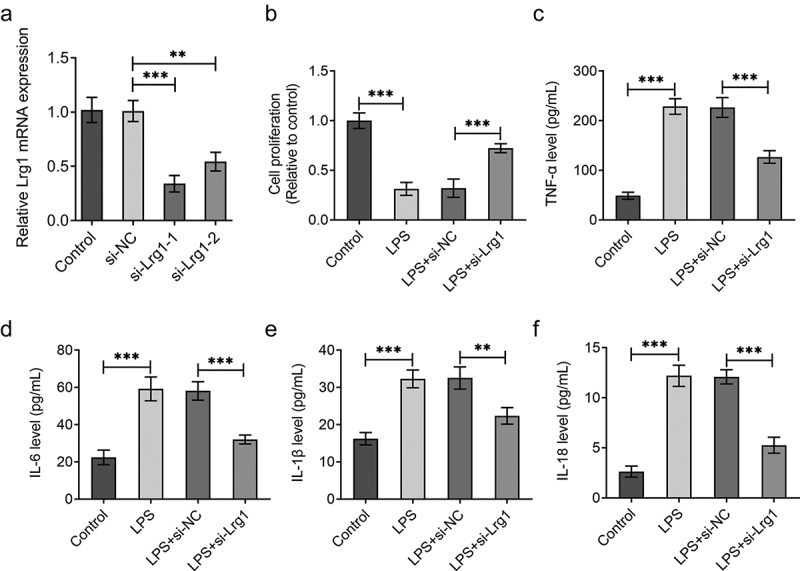
Silencing of Lrg1 ameliorates the inflammation of LPS-induced mouse hippocampal neuronal cells. (a) Lrg1 expression was evaluated by RT-qPCR. (b) Cell viability was detected using a CCK-8 assay. ELISA analysis was to test the levels of inflammatory factors (c) TNF-α, (d) IL-6, (e) IL-1β and (f) IL-18. **P<0.01, ***P<0.001.

**Figure 3.**
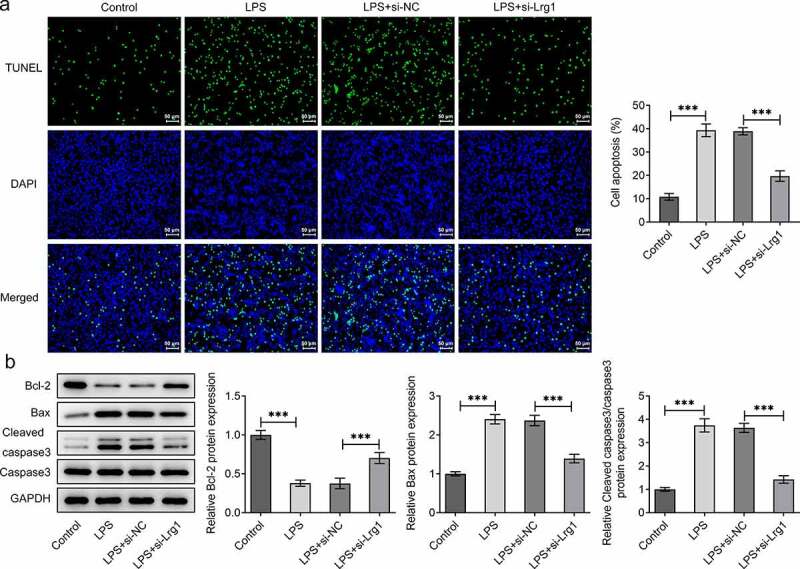
Silencing of Lrg1 ameliorates the apoptosis of LPS-induced mouse hippocampal neuronal cells. (a) TUNEL assay was to detect cell apoptosis. (Magnification, x200). (b) Western blot was to analyze the protein levels of apoptosis-related factors. ***P<0.001.

The function of Lrg1 on the phenotypes of mouse hippocampal neuronal cells treated with LPS was evaluated by subsequent functional experiments. First of all, Lrg1 was silenced by transfection with si-Lrg1-1 and si-Lrg1-2 in HT22 cells, and Lrg1 expression was tested by RT-qPCR. As shown in Figure 2(a), si-Lrg1 expression was significantly downregulated in both si-Lrg1-1 and si-Lrg1-2 groups. HT22 cells transfected with si-Lrg1-1 was selected to perform the subsequent experiments. CCK-8 assay elucidated that LPS treatment apparently suppressed the cell viability while the knockdown of Lrg1 elevated it in LPS-induced HT22 cells (Figure 2(b)). In addition, the enhanced levels of TNF-α, IL-6, IL-1β and IL-18 induced by LPS were all reduced when Lrg1 was down-regulated (Figure 2(c-f)). Following, cell apoptosis was assessed by TUNEL assay and western blot analysis. From the experimental results of TUNEL assay, it was noticed that LPS-stimulated cell apoptosis was abrogated by silencing of Lrg1 (Figure 3(a)). Similarly, LPS treatment lessened the protein level of Bcl-2 but elevated the protein levels of Bax and cleaved caspase3/caspase 3, which were then restored by Lrg1 inhibition (Figure 3(B)). In summary, Lrg1 contributed to the inflammation and apoptosis of LPS-induced HT22 cells.

## Inhibition of Lrg1 inactivates the TGFβ1/SMAD signaling pathway in LPS-induced HT22 cells

**Figure 4.**
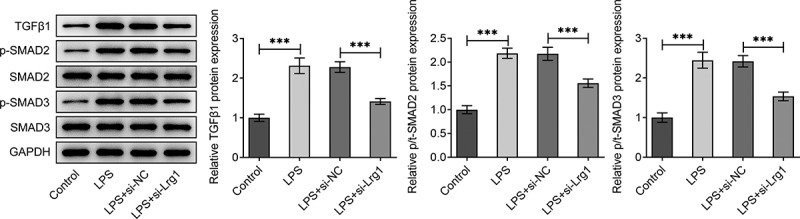
Inhibition of Lrg1 inactivates the TGFβ1/SMAD signaling pathway in LPS-stimulated HT22 cells. Western blot was to analyze the protein levels of related factors in TGFβ1/SMAD signaling pathway. ***P<0.001.

It is reported that Lrg1 is an important activator of TGFβ1/SMAD signaling pathway [23]. Hence, we made a conjecture that Lrg1 might also participate in SAE via the modulation of TGFβ1/SMAD signaling pathway. Through western blot analysis, it was discovered that the enhanced protein levels of TGFβ1, p/t SMAD2 and p/t SMAD3 in HT22 cells upon LPS exposure were all cut down by down-regulation of Lrg1 (Figure 4). In a word, Lrg1 silencing inactivated the TGFβ1/SMAD signaling pathway in HT22 cells under LPS condition.

## Lrg1 silencing attenuates LPS-induced inflammation and apoptosis of HT22 cells through inhibition of TGFβ1/SMAD signaling pathway

**Figure 5.**
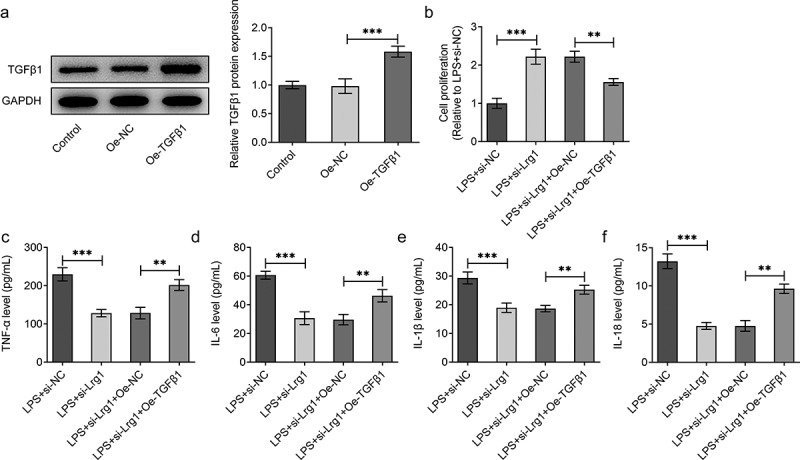
Lrg1 silencing attenuates LPS-induced inflammation of HT22 cells through inhibition of TGFβ1/SMAD signaling pathway. (a) Western blot was to analyze the overexpression efficiency of Lrg1. (b) CCK-8 assay was to evaluate cell viability. ELISA analysis was to examine the levels of inflammatory factors (c) TNF-α, (d) IL-6, (e) IL-1β and (f) IL-18. **P<0.01, ***P<0.001.

**Figure 6.**
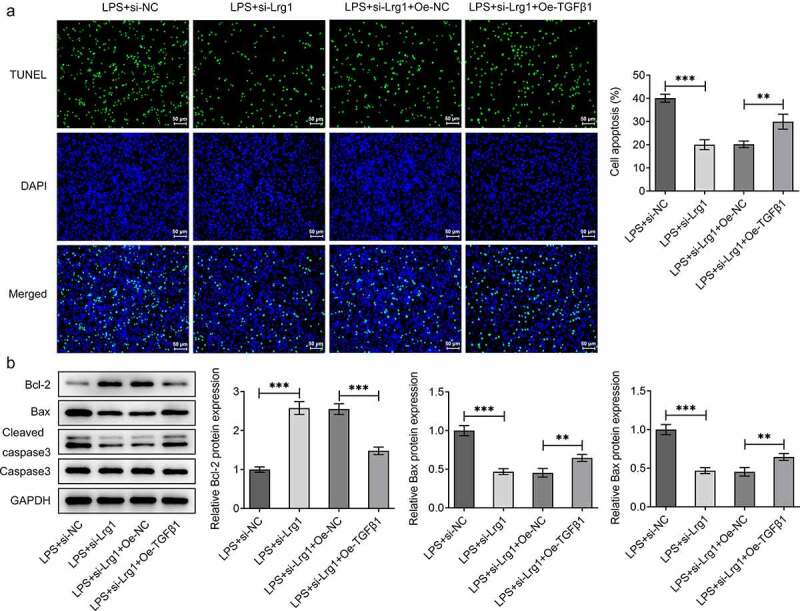
Lrg1 silencing attenuates LPS-induced apoptosis of HT22 cells through inhibition of TGFβ1/SMAD signaling pathway. (A) TUNEL assay was to detect cell apoptosis. (Magnification, x200). (B) Western blot was to analyze the protein levels of apoptosis-related factors. **P<0.01, ***P<0.001.

To confirm the mechanism of Lrg1/TGFβ1/SMAD signaling pathway in SAE, the expression of TGFβ1 was firstly enhanced by transfection of Oe-TGFβ1 plasmid and the overexpression efficiency was tested through western blot analysis (Figure 5(a)). Through CCK-8 assay, Lrg1 deficiency impeded the viability of LPS-induced HT22 cells while this effect was rescued by overexpression of TGFβ1 (Figure 5(b)). On the contrary, the reduced levels of TNF-α, IL-6, IL-1β and IL-18 in HT22 cells caused by Lrg1 knockdown upon LPS exposure were all restored by TGFβ1 up-regulation (Figure 5(c-f)), implying that Lrg1 mediated the inflammatory response of LPS-induced mouse hippocampal neuronal cells via the modulation of TGFβ1/SMAD signaling pathway. Likewise, the results from TUNEL assay showed that TGFβ1 overexpression offset the suppressed apoptosis of LPS-treated HT22 cells due to Lrg1 silencing (Figure 6(a)). Also, Lrg1 abrogation elevated the protein level of Bcl-2 and decreased the protein levels of Bax and cleaved caspase 3/caspase 3 in LPS-stimulated HT22 cells while this effect was counteracted by up-regulation of TGFβ1 (Figure 6(b)). Taken together, Lrg1 silencing relieved LPS-induced inflammation and apoptosis of HT22 cells through inhibition of TGFβ1/SMAD signaling pathway.

## Discussion

SAE is defined as a common and fatal complication of sepsis which may result in poor outcomes including neuroinflammation, long-lasting mental impairment, cognitive impairment [^24–26^]. Accumulating studies have suggested that neuroinflammation is emerging as a key player of brain dysfunction in sepsis [27]. TNF- α, a proinflammatory cytokine, is associated with several neurological disorders [28]. IL-6 is a cytokine associated with both immune response and neurogenesis [29]. Also, IL-1β has been implicated in immune responses and CNS-related diseases [30]. IL-18 is a powerful inflammatory cytokine of the IL-1 family and mediates neuroinflammation and neurodegeneration in CNS [31]. In line with this, the experimental results from ELISA analysis underlined that sepsis might lead to inflammatory response by elevating the levels of TNF-α, IL-6, IL-1β and IL-18. Also, OFT and MWM in our study demonstrated that sepsis mice exhibited anxiety-like behaviors and cognitive impairment. Besides, H&E staining also determined that sepsis causes neuronal changes in mice. All above results indicated the successful establishment of sepsis-induced brain damage model in mice.

LPS is generally deemed as a unique family of glycolipids based on a highly conserved lipid moiety [32]. The cellular functions in different cell types vary in response to LPS [33]. More intriguingly, the modification of LPS is of great significance to the development of live attenuated vaccines for the reason that LPS is a component of the cell wall of gram-negative bacteria [34]. It is also known as a major microbial mediator in the pathogenesis of sepsis [35,36]. LPS is also well documented to trigger powerful immune responses and can even initiate symptoms of sepsis [37]. Hence, LPS was utilized to treat mouse hippocampal neuronal cells to stimulate sepsis model in vitro. The experimental results disclosed that LPS led to the decrease in cell viability and the increase in inflammatory response and cell apoptosis, which was in line with the previous studies [19,38].

Lrg1 is a secreted protein implicated in a variety of malignancies [39]. More importantly, Lrg1 has higher serum levels in sepsis patients [14] and has been regarded as a potential new biomarker for sepsis [15]. A previous study has highlighted that Lrg concentration in human cerebrospinal fluid increases with age, or due to neurodegenerative diseases such as Parkinson’s disease with dementia and dementia with Lewy bodies [40]. It is noteworthy that Lrg1 can promote apoptosis and autophagy to exacerbate ischemia/reperfusion-induced brain injury [16]. Lrg1 overexpression in the brain hippocampal contributes to memory impairment [41]. What’s more, LPS treatment enhances the expression of Lrg mRNA in a dose-dependent manner in hepatocytes [42]. The employment of GEO2R analysis elaborated that Lrg1 displayed high expression in brain tissues of SAE mice. This study firstly uncovered that Lrg1 expression was definitely high in brain tissues of sepsis mice. Functional experiments proved that the viability was repressed while the inflammatory response and apoptosis in HT22 cells were both promoted upon LPS exposure, whereas these results could be restored by silencing of Lrg1.

As reported, TGFβ can cross talk with other signaling pathways to mediate multiple biological activities [43]. In TGFβ1/SMAD signaling pathway, TGF-β1 stimulates its downstream Smads to exert its regulatory roles in diverse human diseases [44]. For instance, TGF-β/Smad signaling is an important pathway in the pathogenesis of renal fibrosis and inflammation in chronic kidney disease [45]. Su et al. have clarified that TGFβ1/SMAD pathway is a critical mediator in human chronic myeloid leukemia [46]. Furthermore, the activation of TGFβ1/SMAD pathway has been verified to facilitate sepsis-induced acute lung injury [47]. TGFBR2 overexpression aggravates LPS-induced sepsis through up-regulating Smad2/3 [48]. At the same time, numerous studies have emphasized that Lrg1 is associated with TGF-β/Smad signaling. For example, Lrg1 drives ischemia/reperfusion injury through TGFβ-smad1/5 signaling pathway [16]. Lrg1 stimulates angiogenesis via activating the TGFβ1 signaling [11]. In accordance with this, this study also validated that the protein levels of TGFβ1, p/t SMAD2 and p/t SMAD3 were declined in LPS-induced HT22 cells while this effect was reversed by Lrg1 reduction. Meanwhile, up-regulation of TGFβ1 restored the induced cell proliferation, inhibited inflammatory response and apoptosis on account of Lrg1 knockdown.

## Conclusion

To be concluded, our findings demonstrated that Lrg1 silencing could alleviate brain injury in SAE both *in vitro and in vivo* by inhibiting the TGFβ1/SMAD signaling. This study highlights that Lrg1 might be a promising target for the treatment of SAE.

## Supplementary Material

Supplemental Material

## Data Availability

All data included in this study are available upon request through contact with the corresponding author.
