# Supplementary figures and images for: Leucine rich alpha-2-glycoprotein 1 (Lrg1) silencing protects against sepsis-mediated brain injury by inhibiting transforming growth factor beta1 (TGFβ1)/SMAD signaling pathway
Source: Bioengineered. 2022 Mar 10;13(3):7316–27. doi: 10.1080/21655979.2022.2048775 (PMC8973760; doi:10.1080/21655979.2022.2048775)

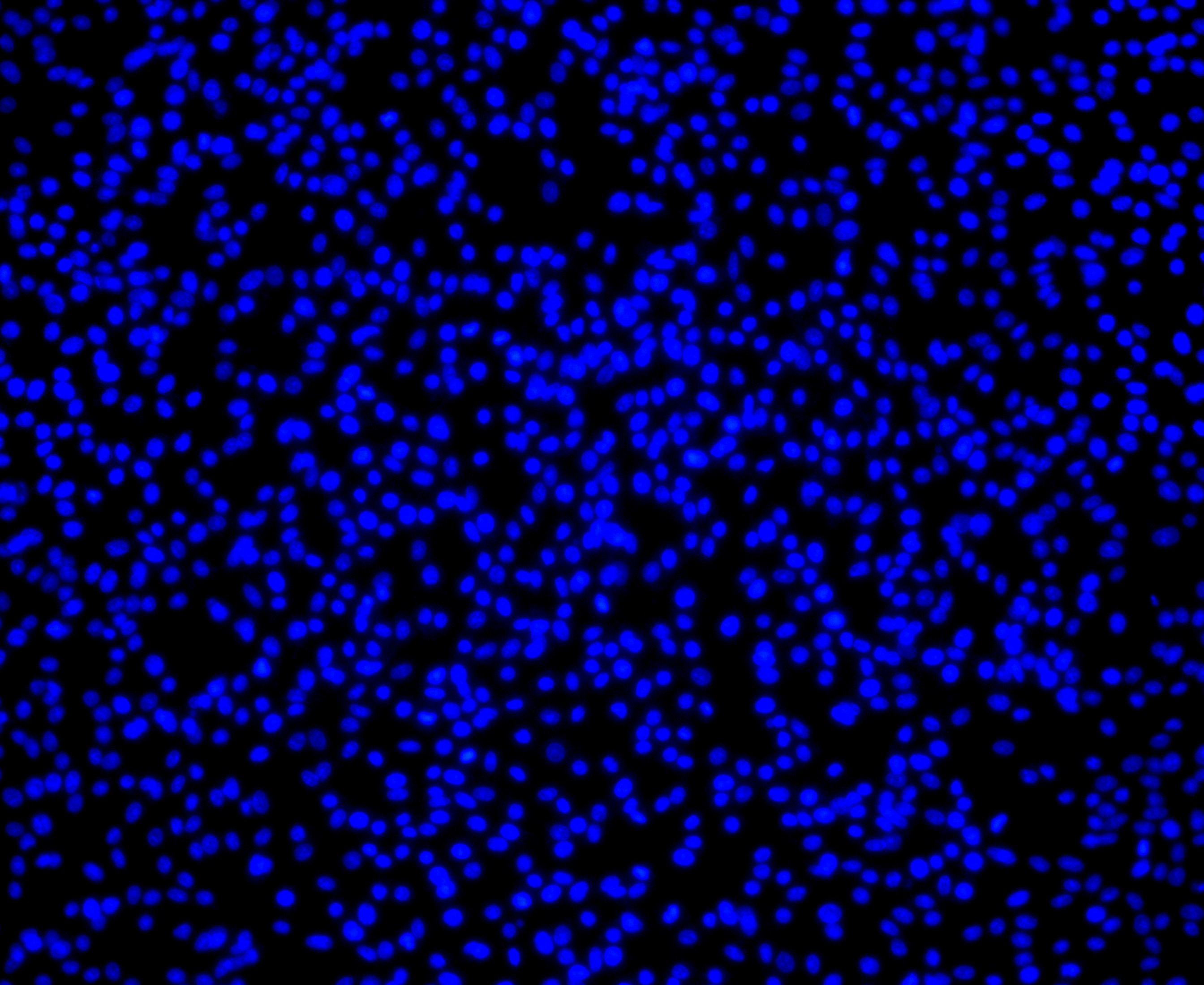

Supplement: Supplemental Material [file KBIE_A_2048775_SM5166.zip › Fig3A_Control_DAPI.tif]

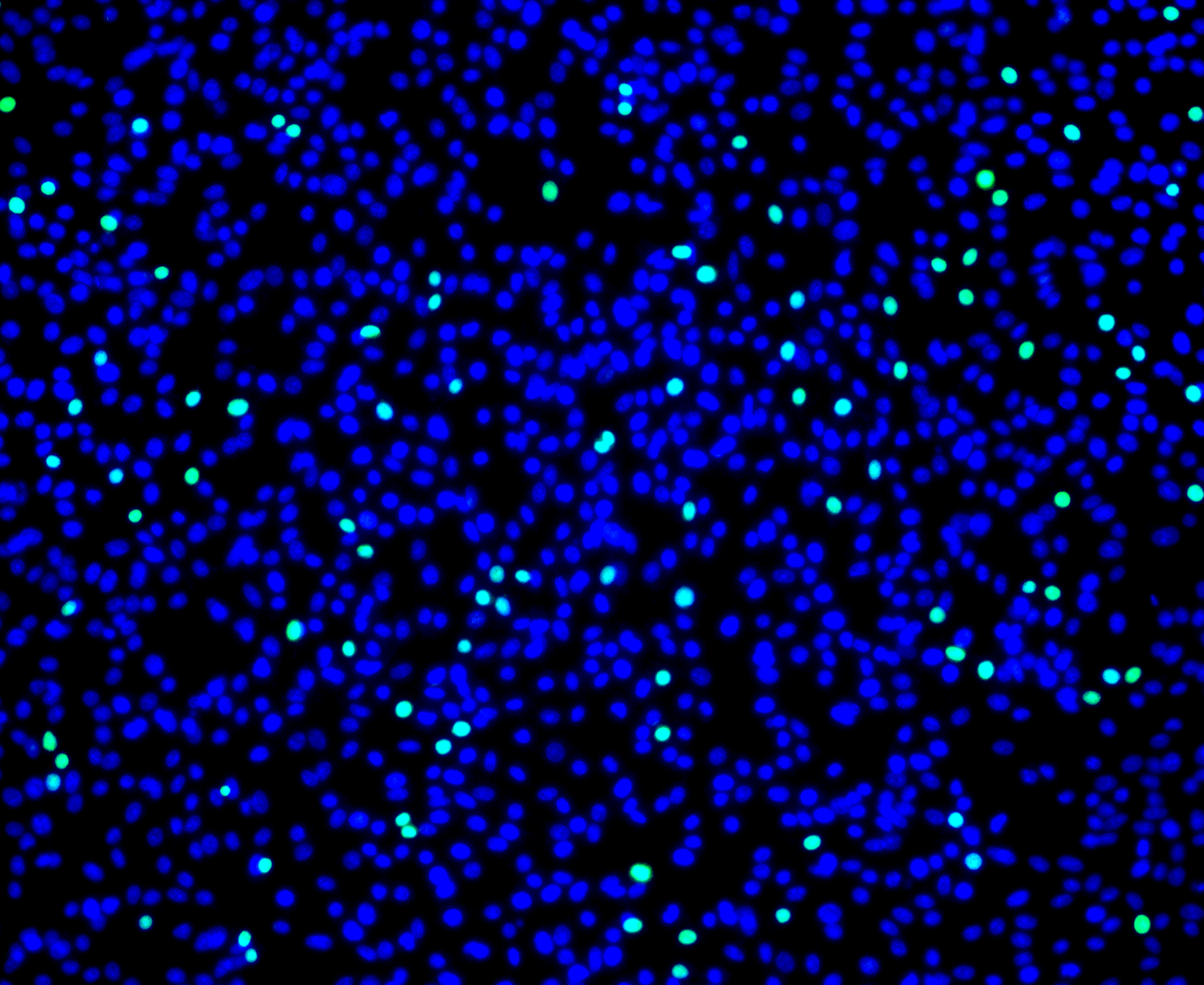

Supplement: Supplemental Material [file KBIE_A_2048775_SM5166.zip › Fig3A_Control_Merged.tif]

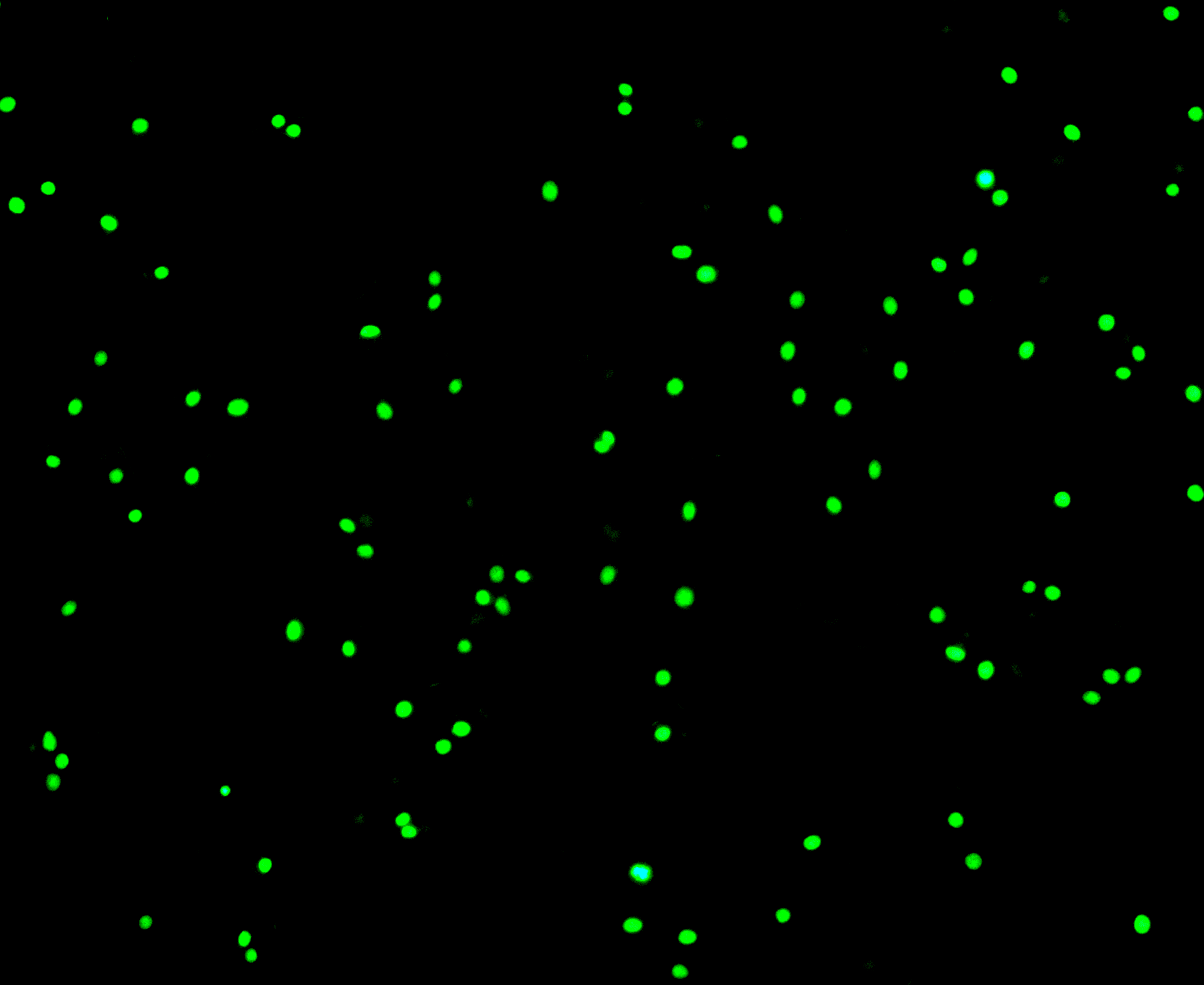

Supplement: Supplemental Material [file KBIE_A_2048775_SM5166.zip › Fig3A_Control_TUNEL.tif]

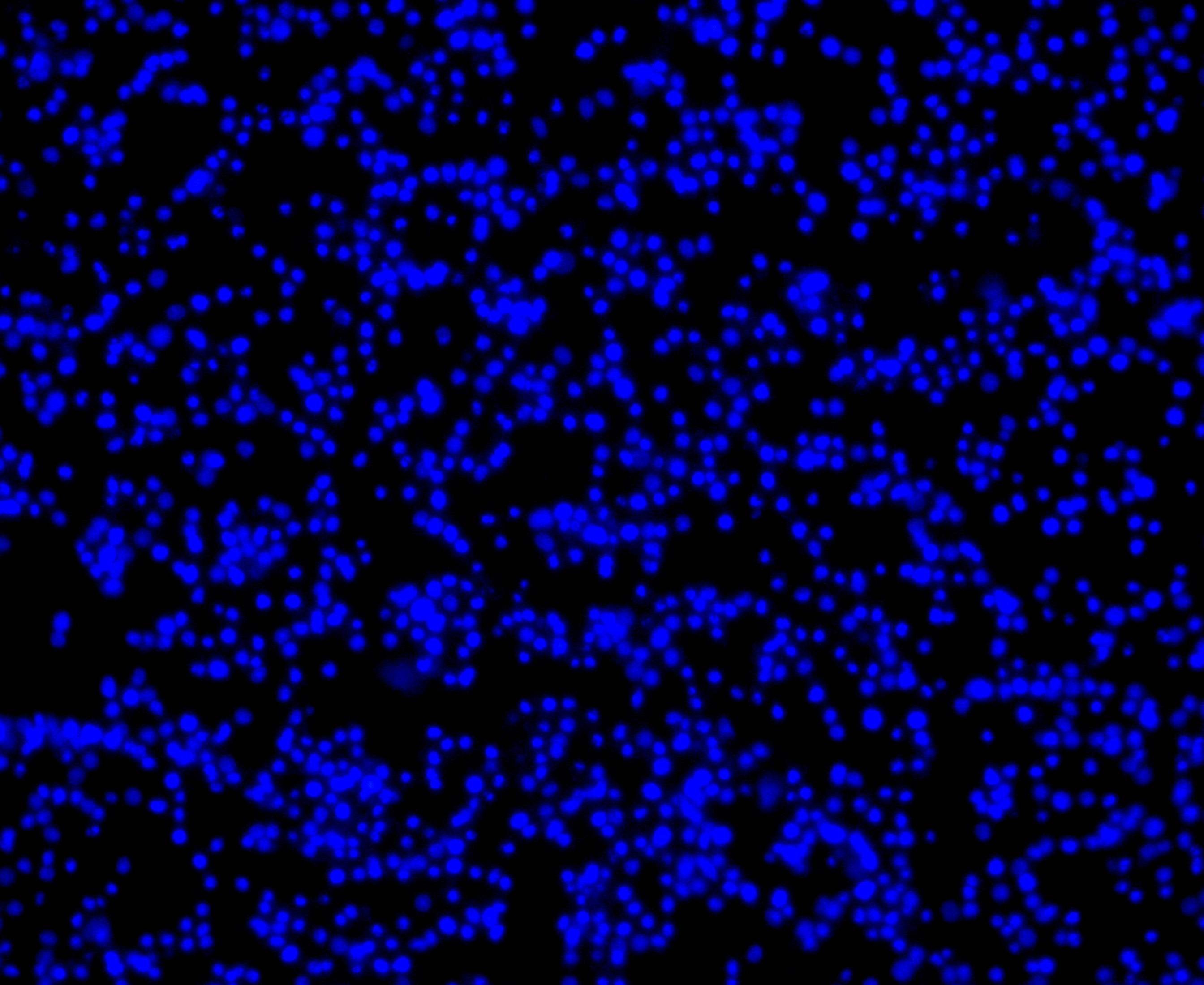

Supplement: Supplemental Material [file KBIE_A_2048775_SM5166.zip › Fig3A_LPS_DAPI.tif]

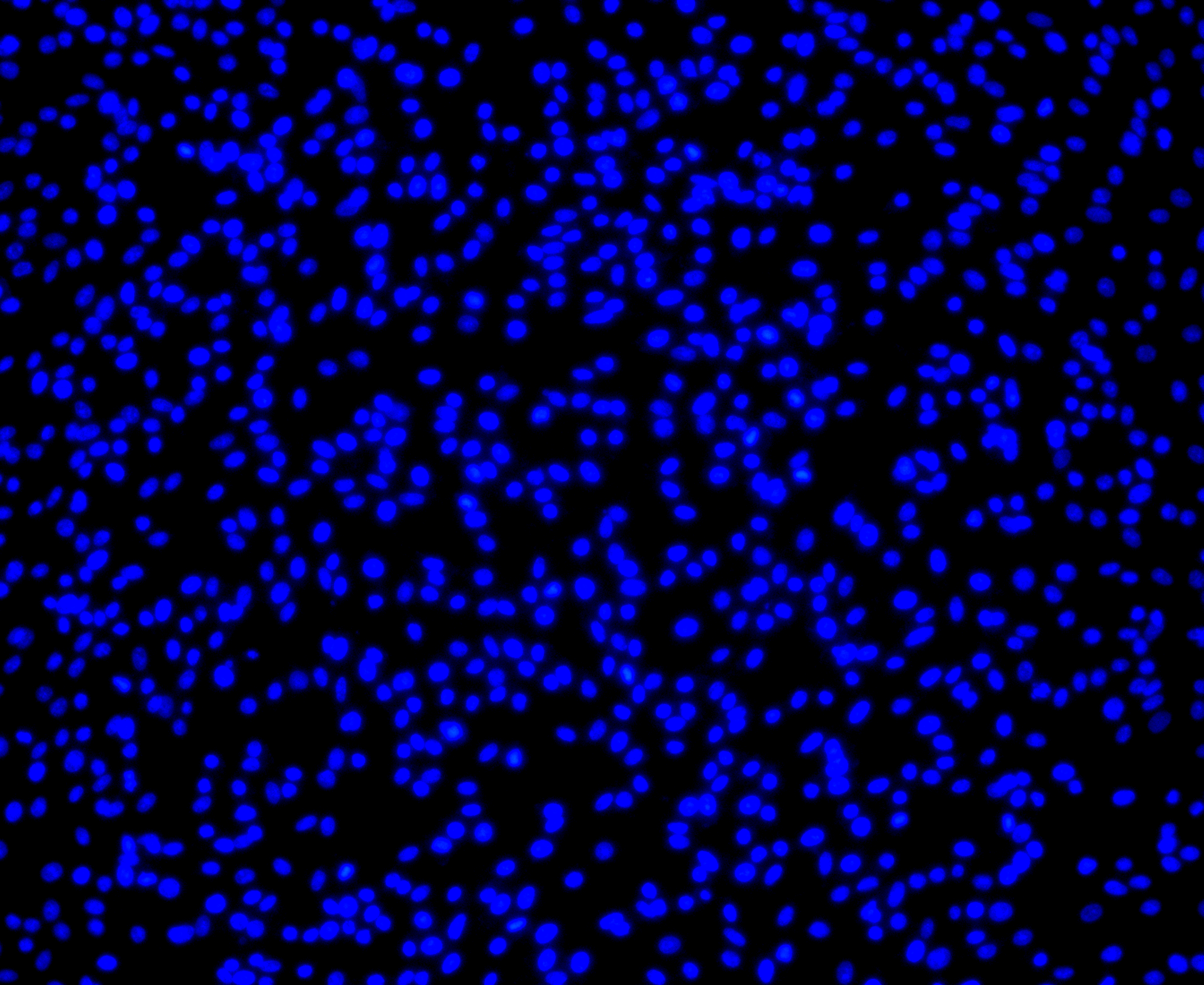

Supplement: Supplemental Material [file KBIE_A_2048775_SM5166.zip › Fig3A_LPS_Si_Lrg1_DAPI.tif]

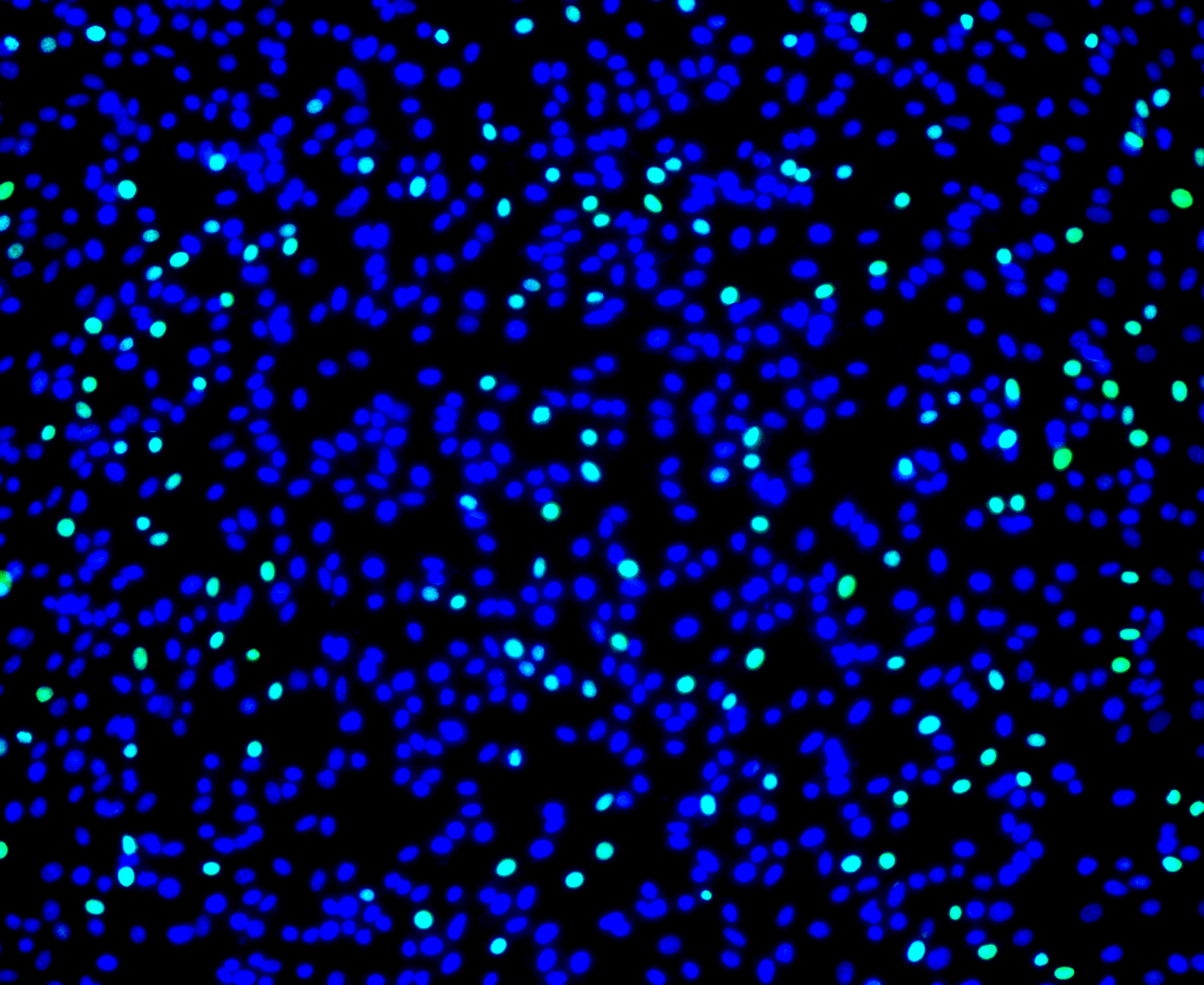

Supplement: Supplemental Material [file KBIE_A_2048775_SM5166.zip › Fig3A_LPS_Si_Lrg1_Merged.tif]

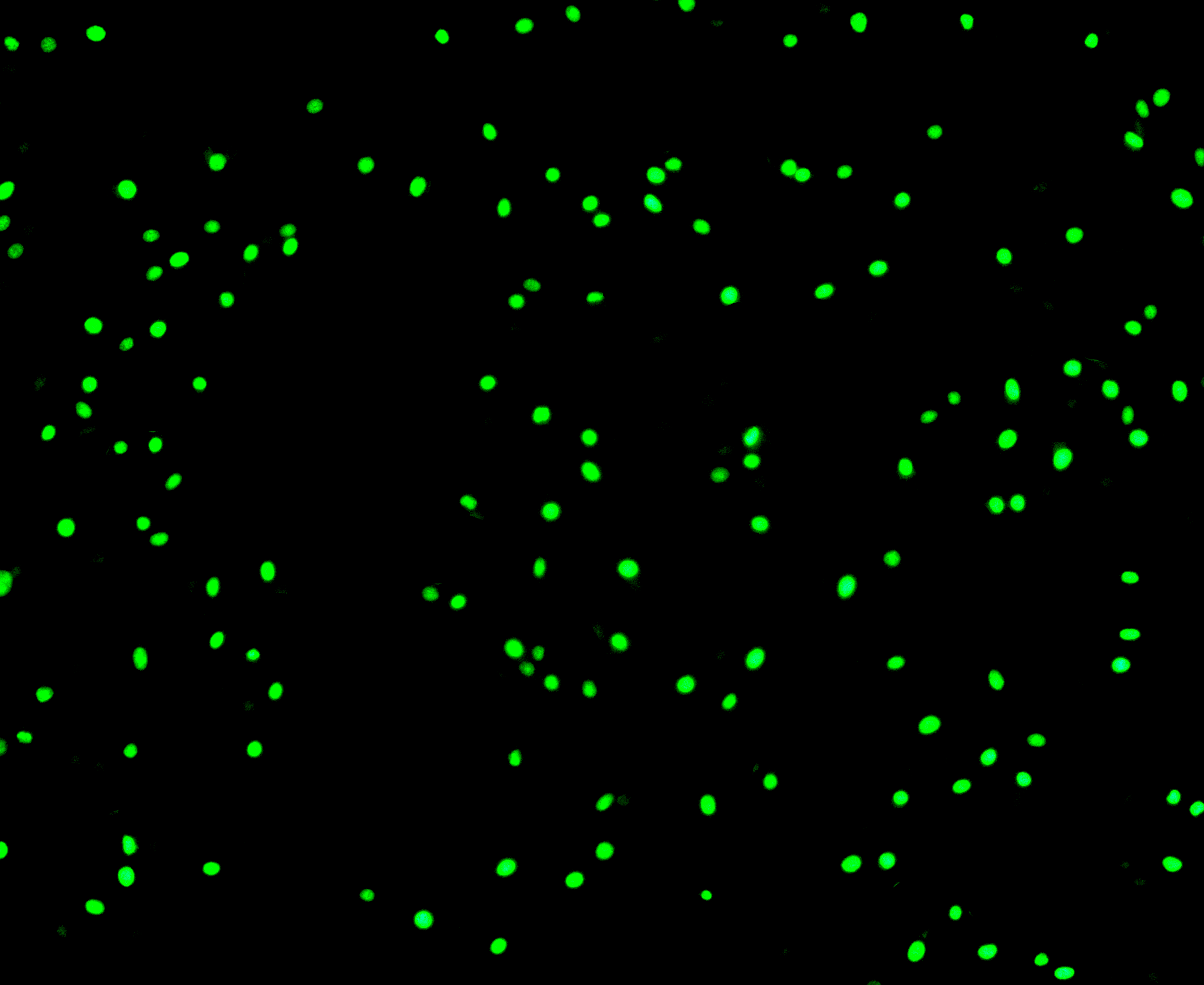

Supplement: Supplemental Material [file KBIE_A_2048775_SM5166.zip › Fig3A_LPS_Si_Lrg1_TUNEL.tif]

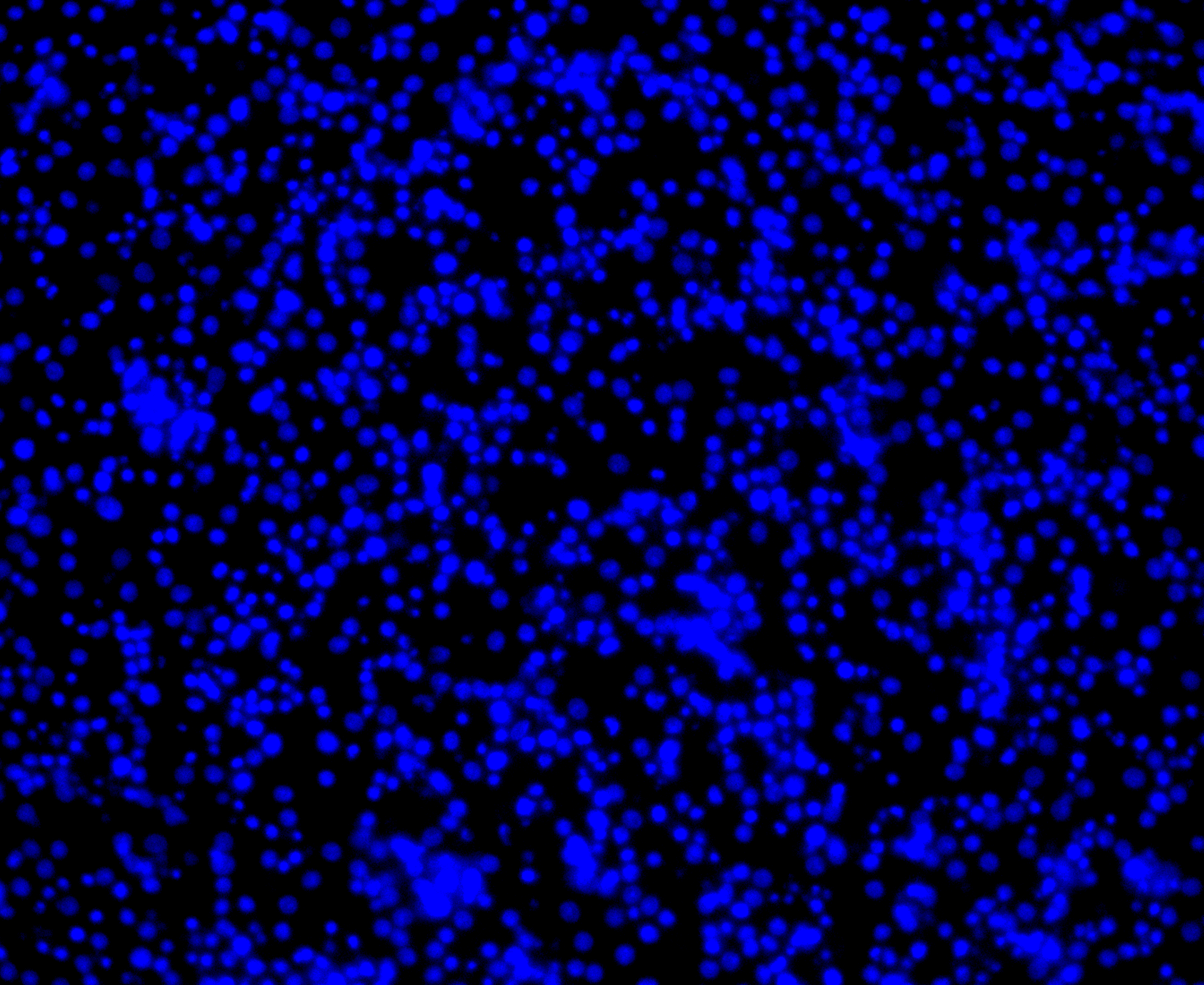

Supplement: Supplemental Material [file KBIE_A_2048775_SM5166.zip › Fig3A_LPS_Si_NC_DAPI.tif]

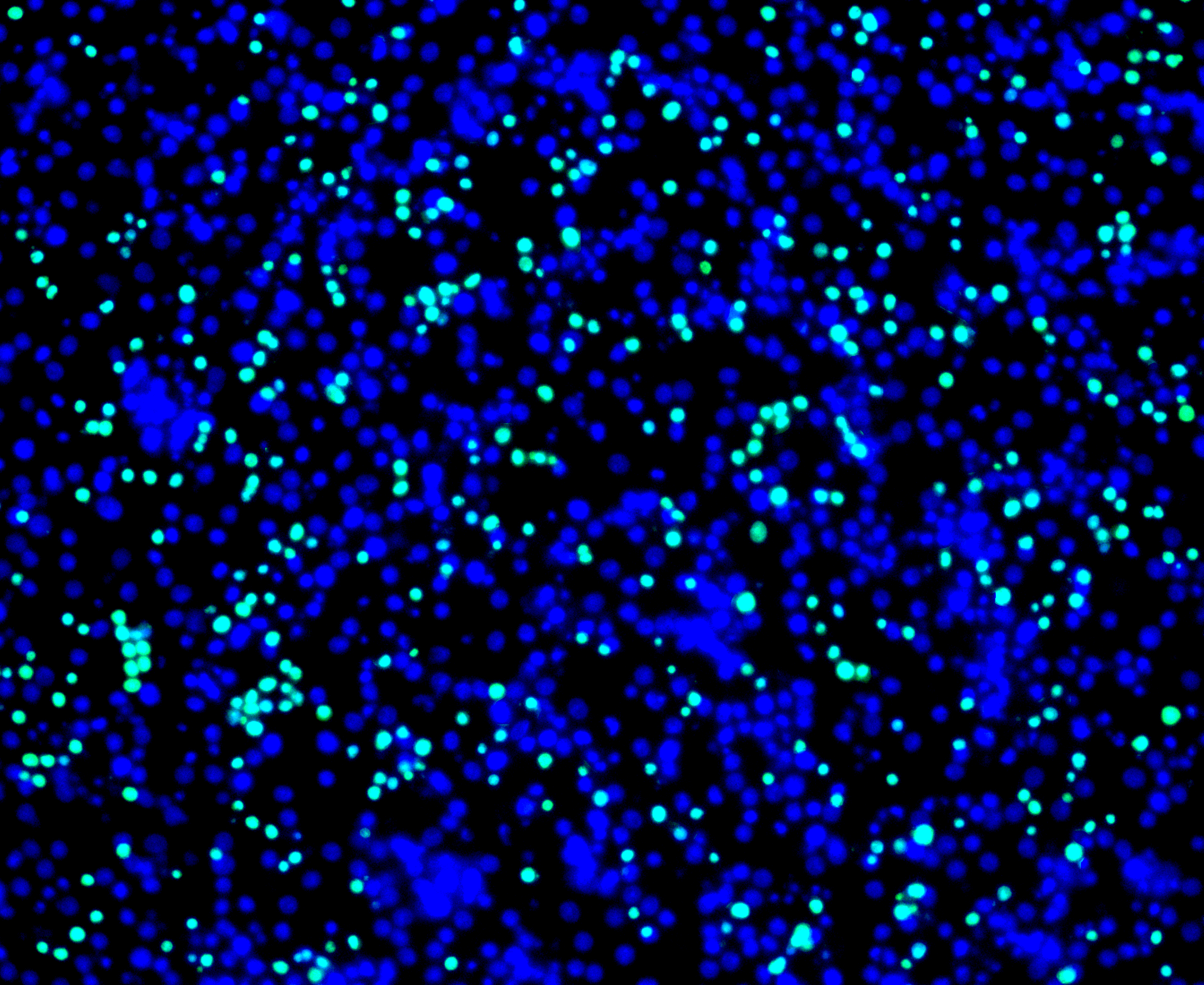

Supplement: Supplemental Material [file KBIE_A_2048775_SM5166.zip › Fig3A_LPS_Si_NC_Merged.tif]

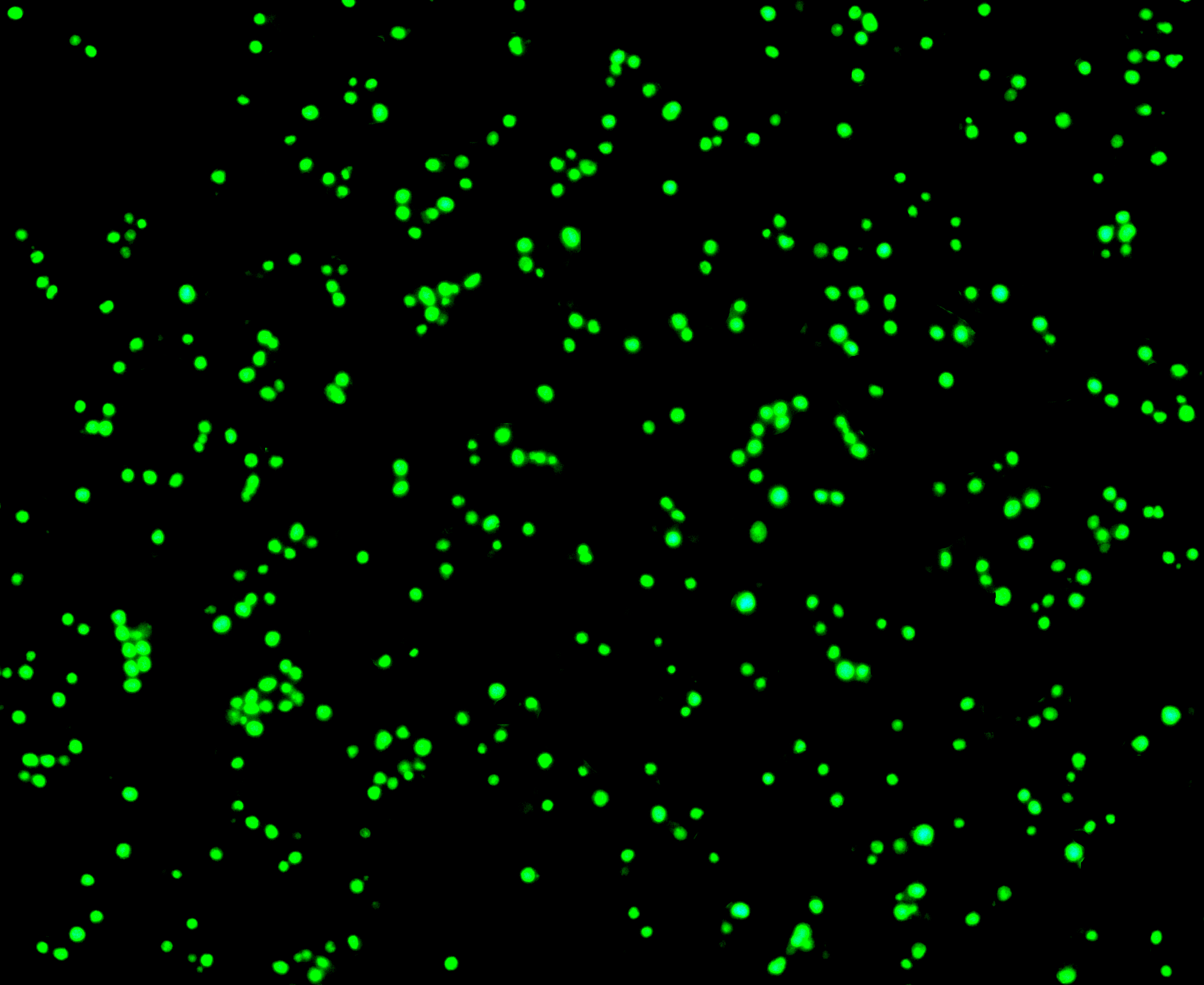

Supplement: Supplemental Material [file KBIE_A_2048775_SM5166.zip › Fig3A_LPS_Si_NC_TUNEL.tif]

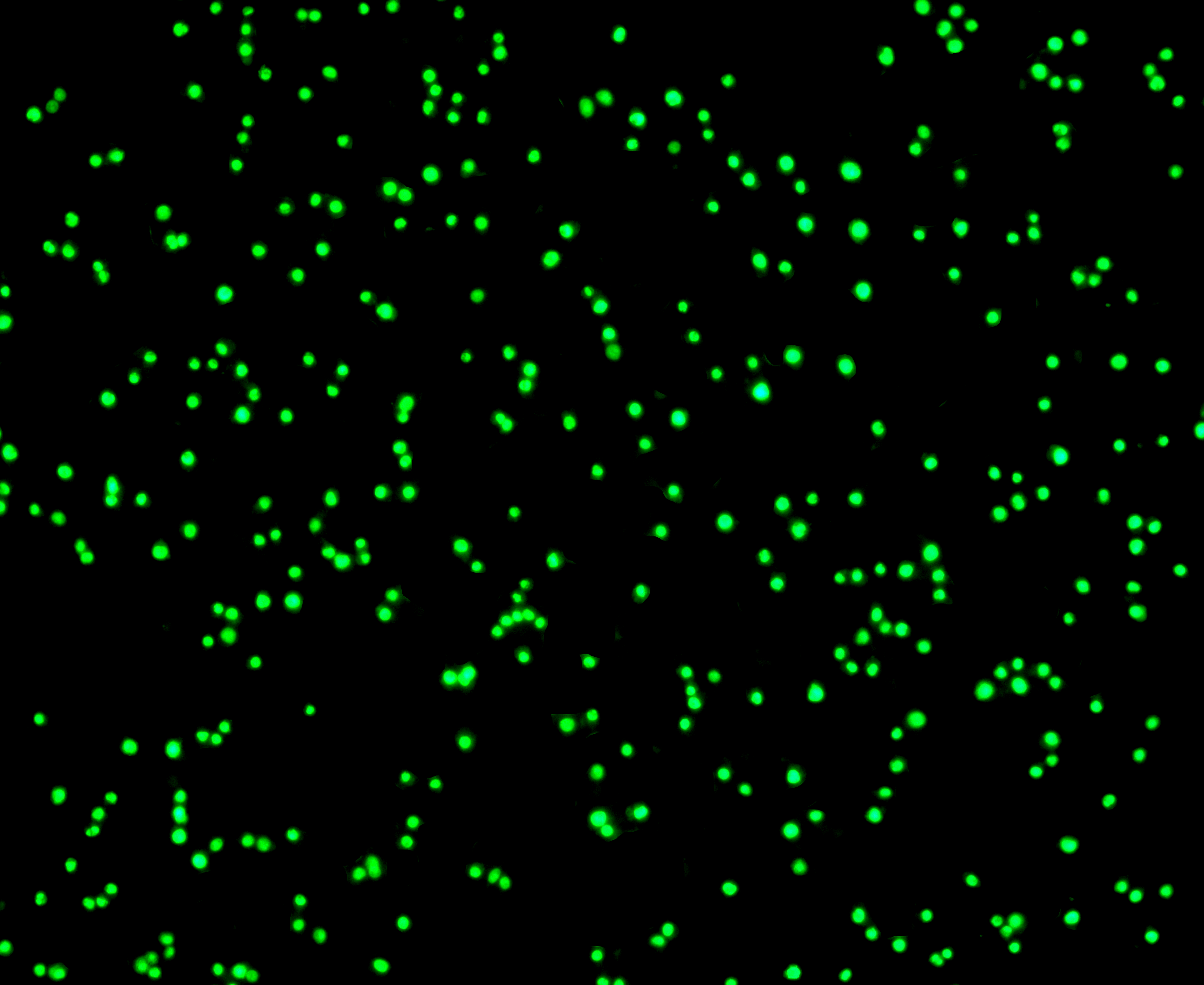

Supplement: Supplemental Material [file KBIE_A_2048775_SM5166.zip › Fig3A_LPS_TUNEL.tif]

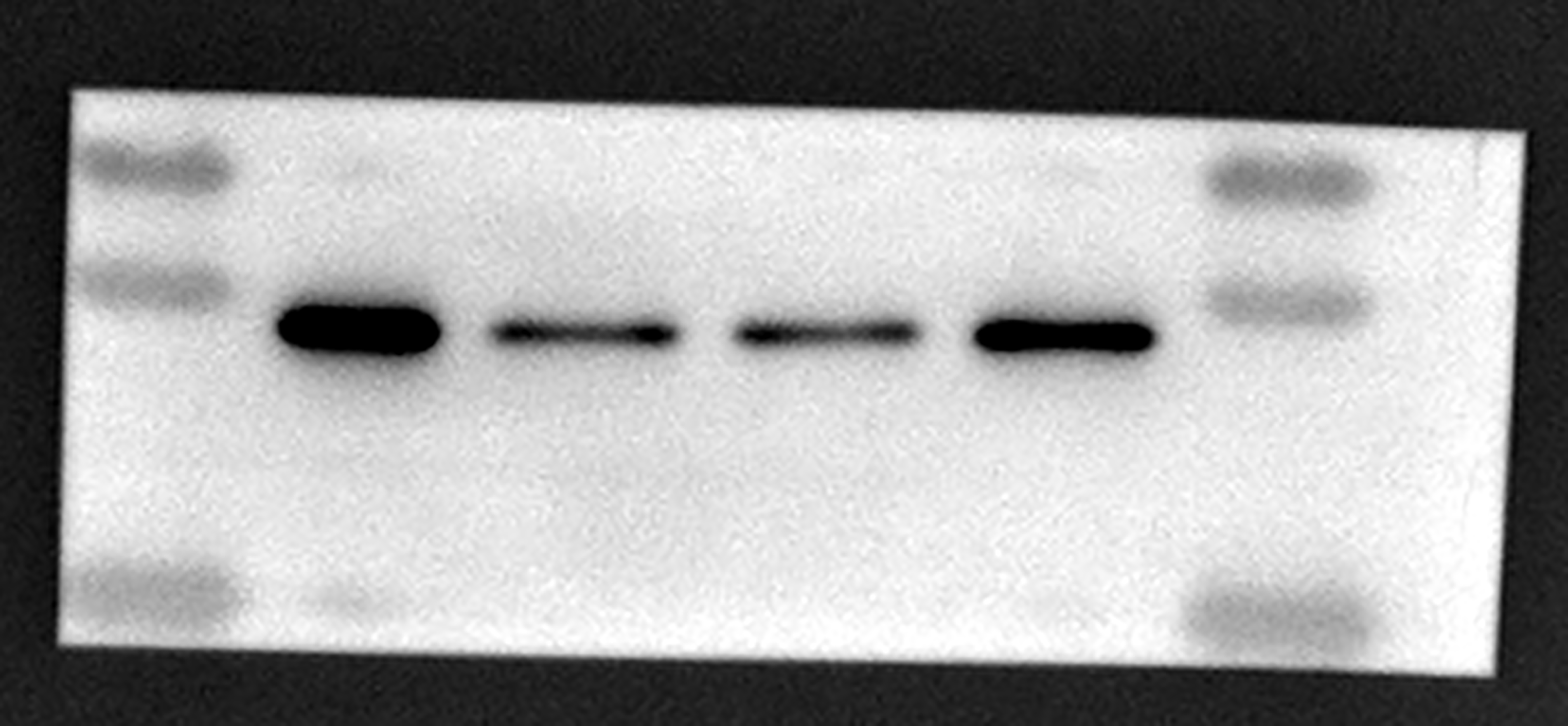

Supplement: Supplemental Material [file KBIE_A_2048775_SM5166.zip › Fig3B_Bcl_2.tif]

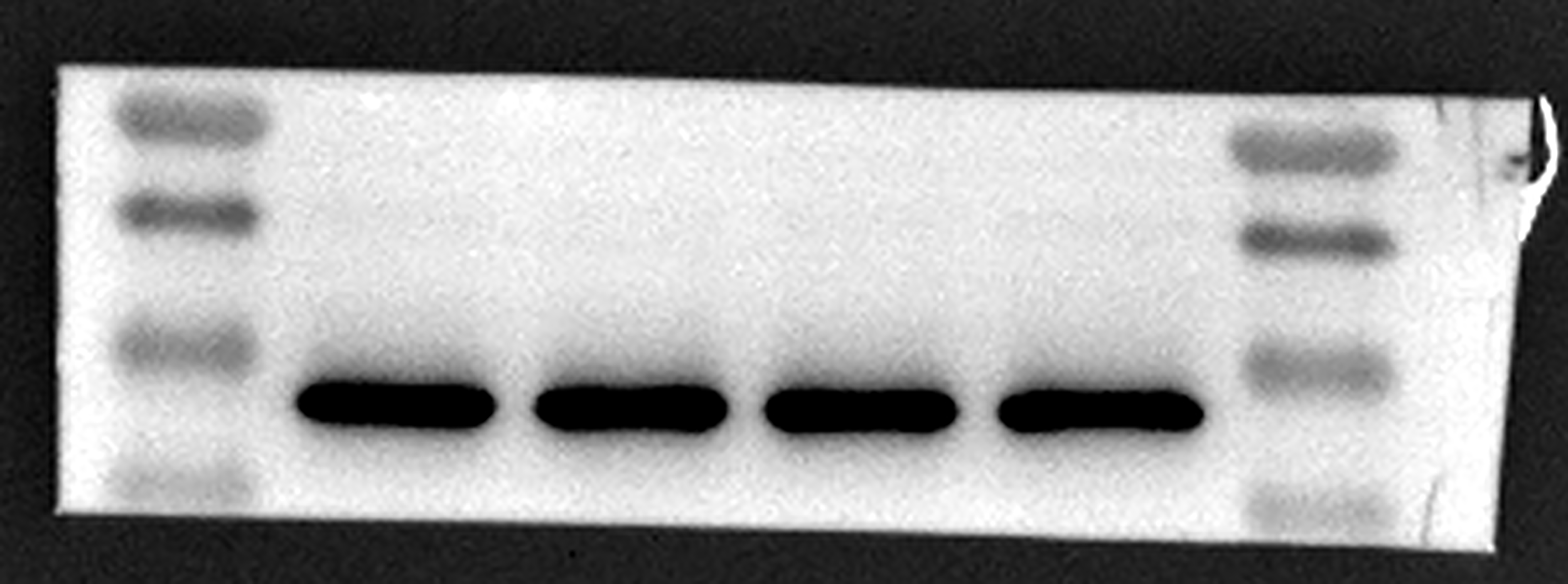

Supplement: Supplemental Material [file KBIE_A_2048775_SM5166.zip › Fig3B_caspase3.tif]

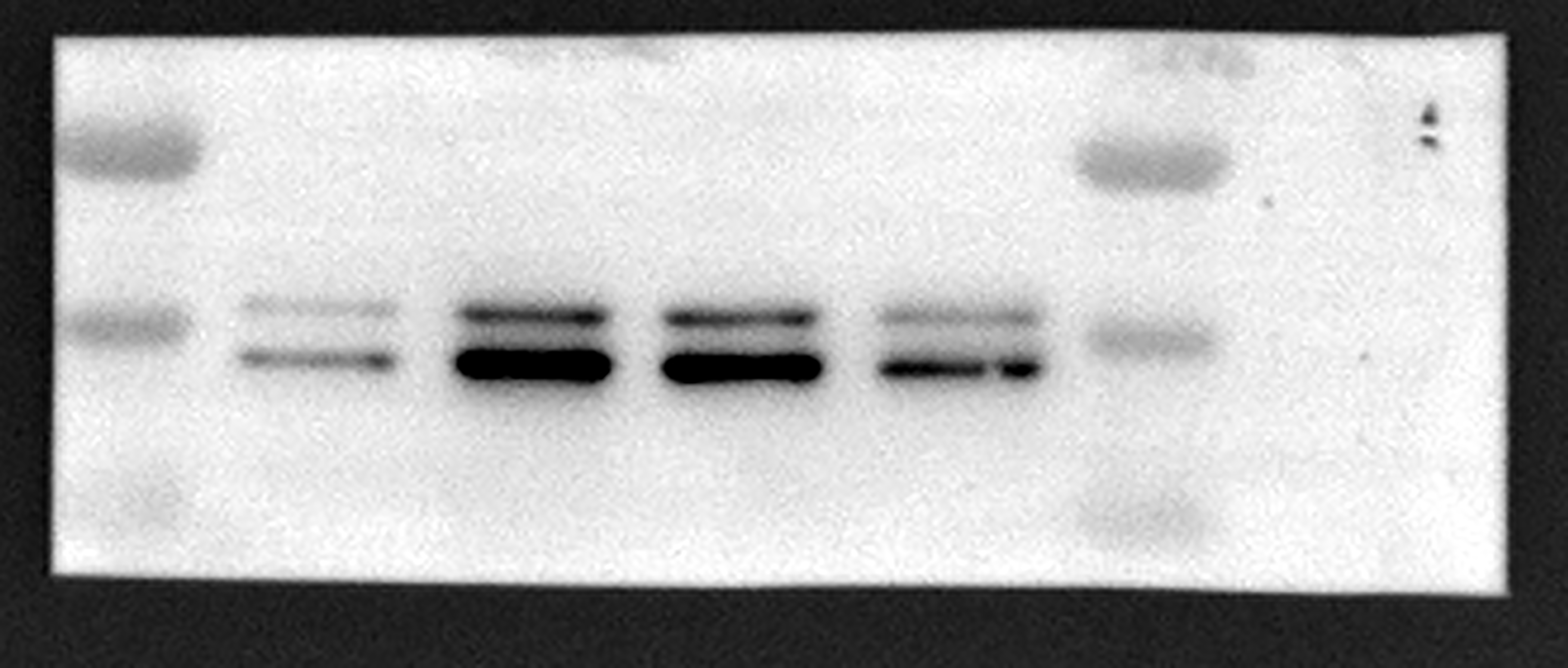

Supplement: Supplemental Material [file KBIE_A_2048775_SM5166.zip › Fig3B_cleaved caspase3.tif]

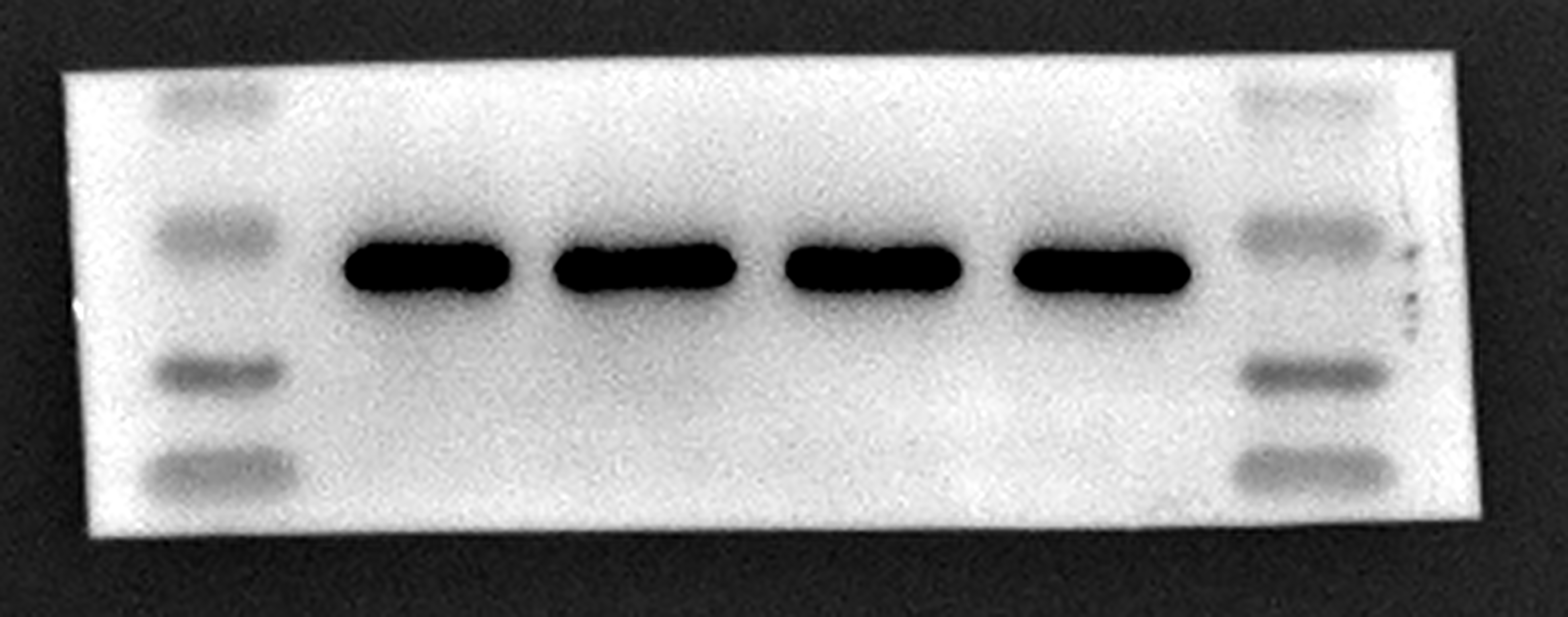

Supplement: Supplemental Material [file KBIE_A_2048775_SM5166.zip › Fig3B_GAPDH.tif]

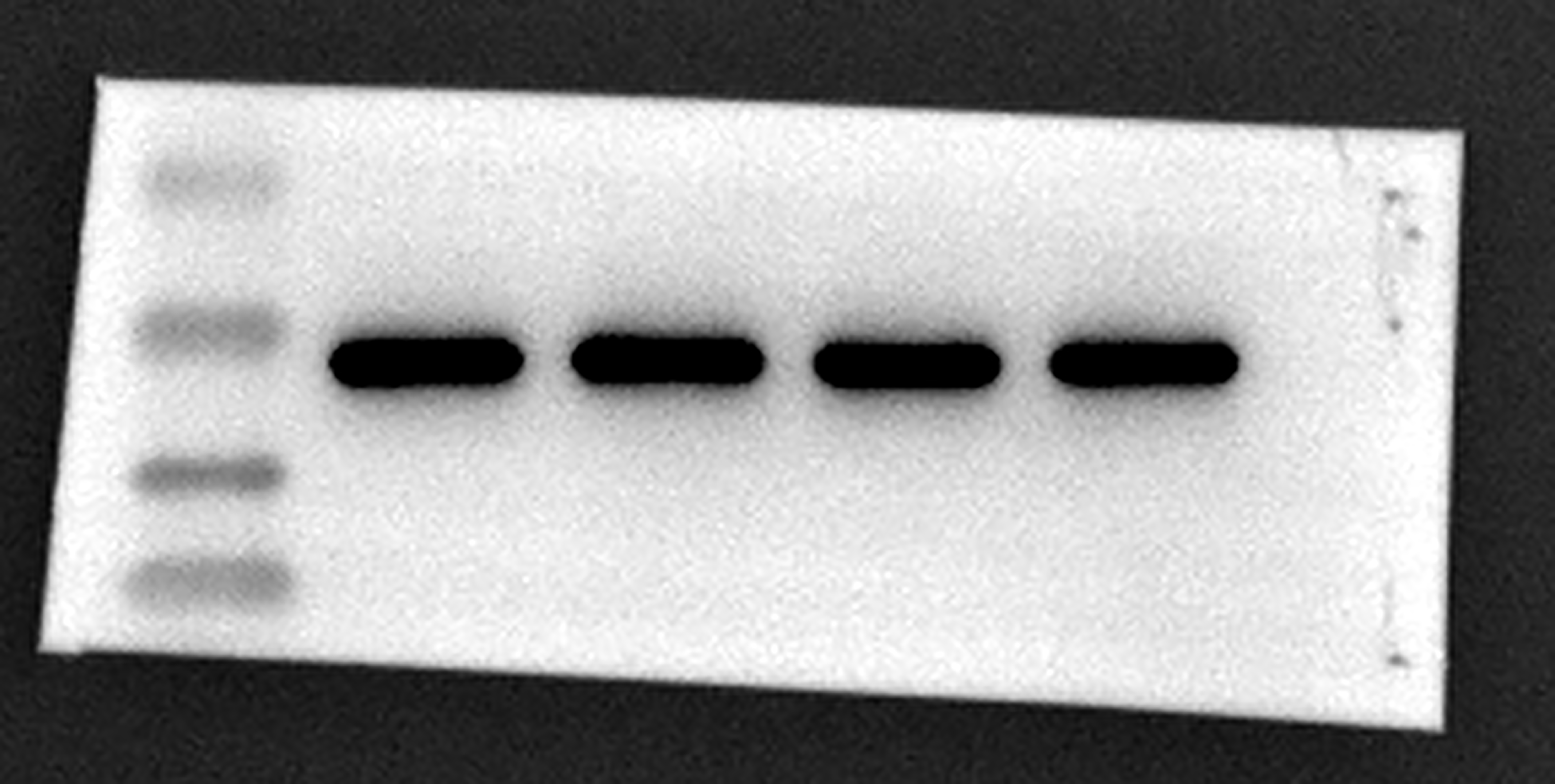

Supplement: Supplemental Material [file KBIE_A_2048775_SM5166.zip › Fig4_GAPDH.tif]

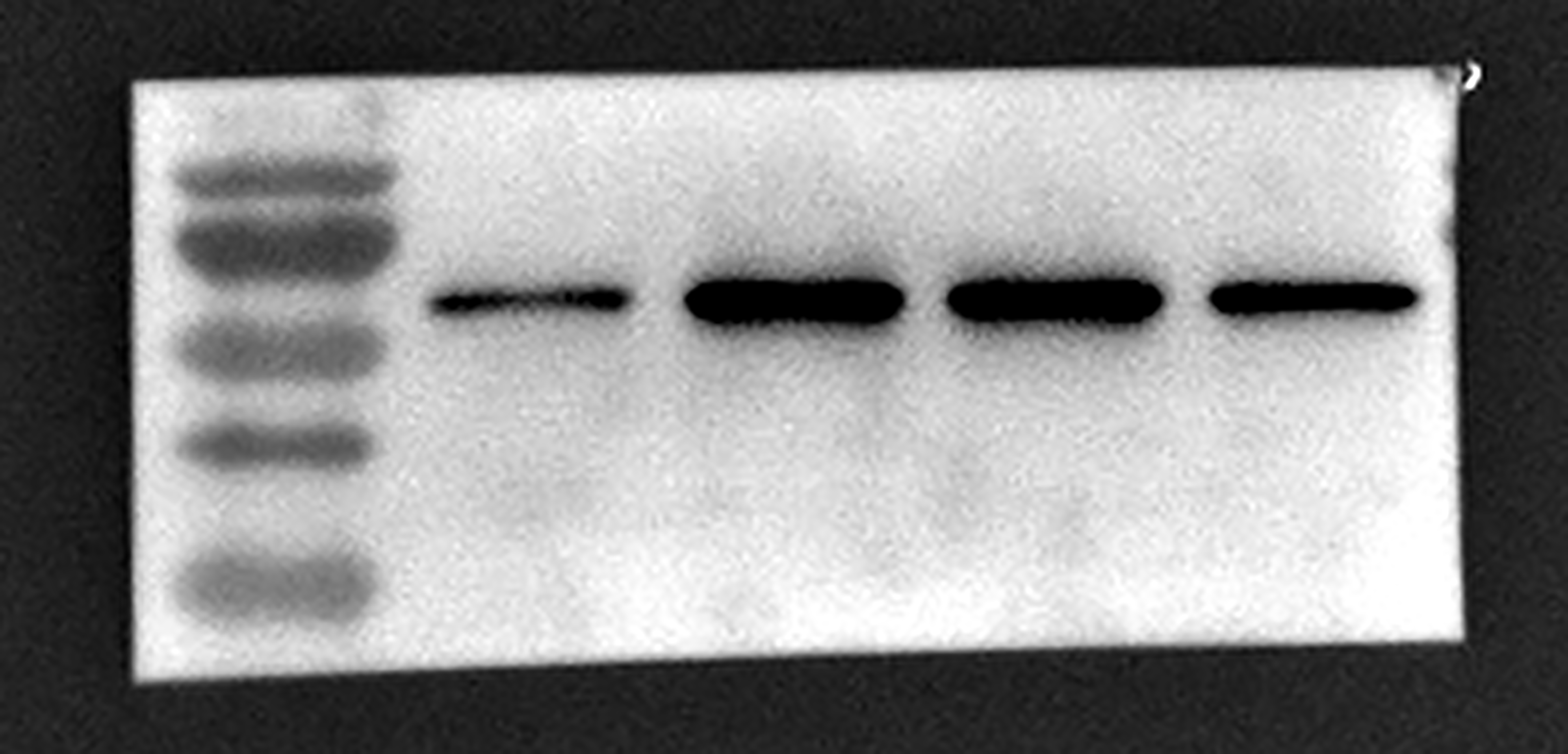

Supplement: Supplemental Material [file KBIE_A_2048775_SM5166.zip › Fig4_p_SMAD3.tif]

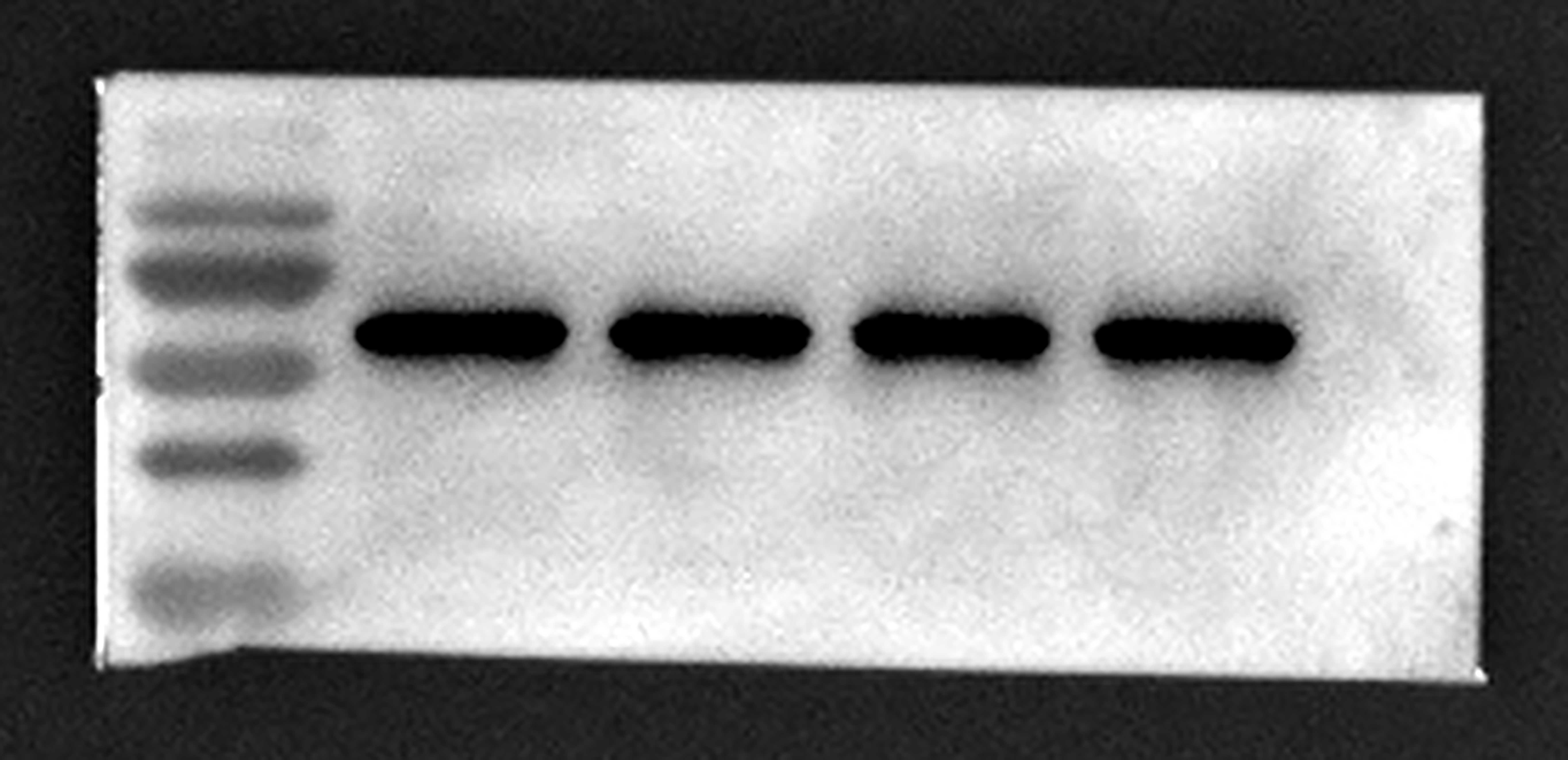

Supplement: Supplemental Material [file KBIE_A_2048775_SM5166.zip › Fig4_SMAD2.tif]

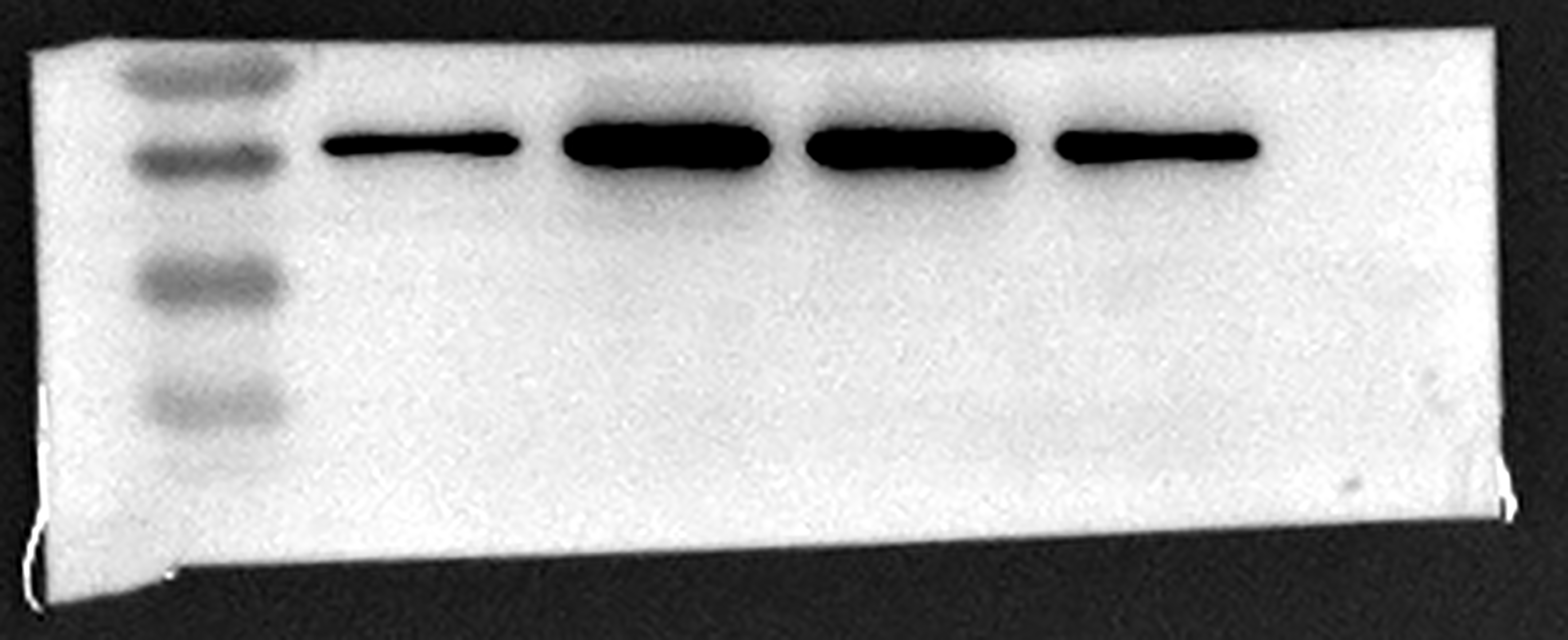

Supplement: Supplemental Material [file KBIE_A_2048775_SM5166.zip › Fig4_TGFb1.tif]

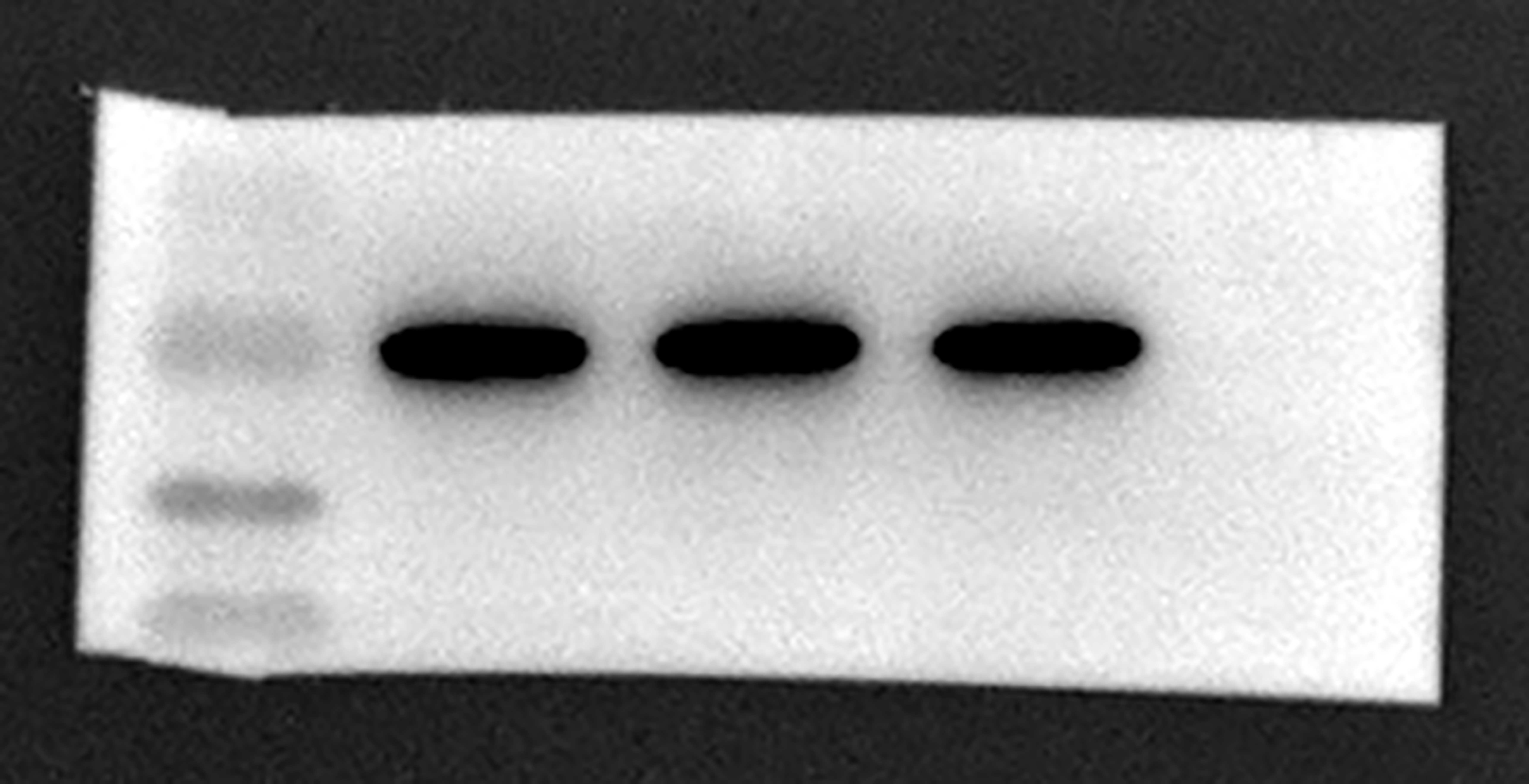

Supplement: Supplemental Material [file KBIE_A_2048775_SM5166.zip › Fig5A_GAPDH.tif]

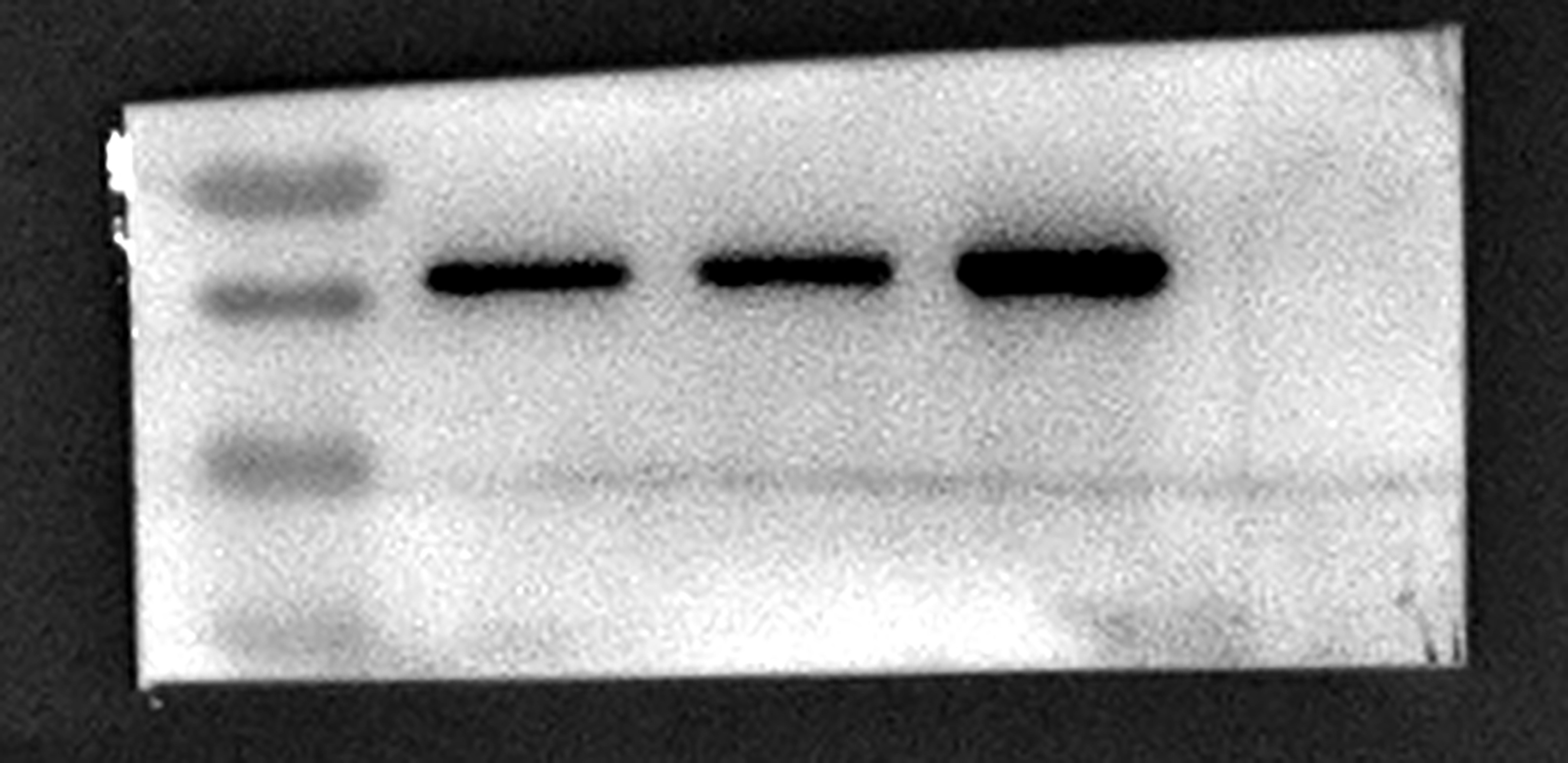

Supplement: Supplemental Material [file KBIE_A_2048775_SM5166.zip › Fig5A_TGFb1.tif]

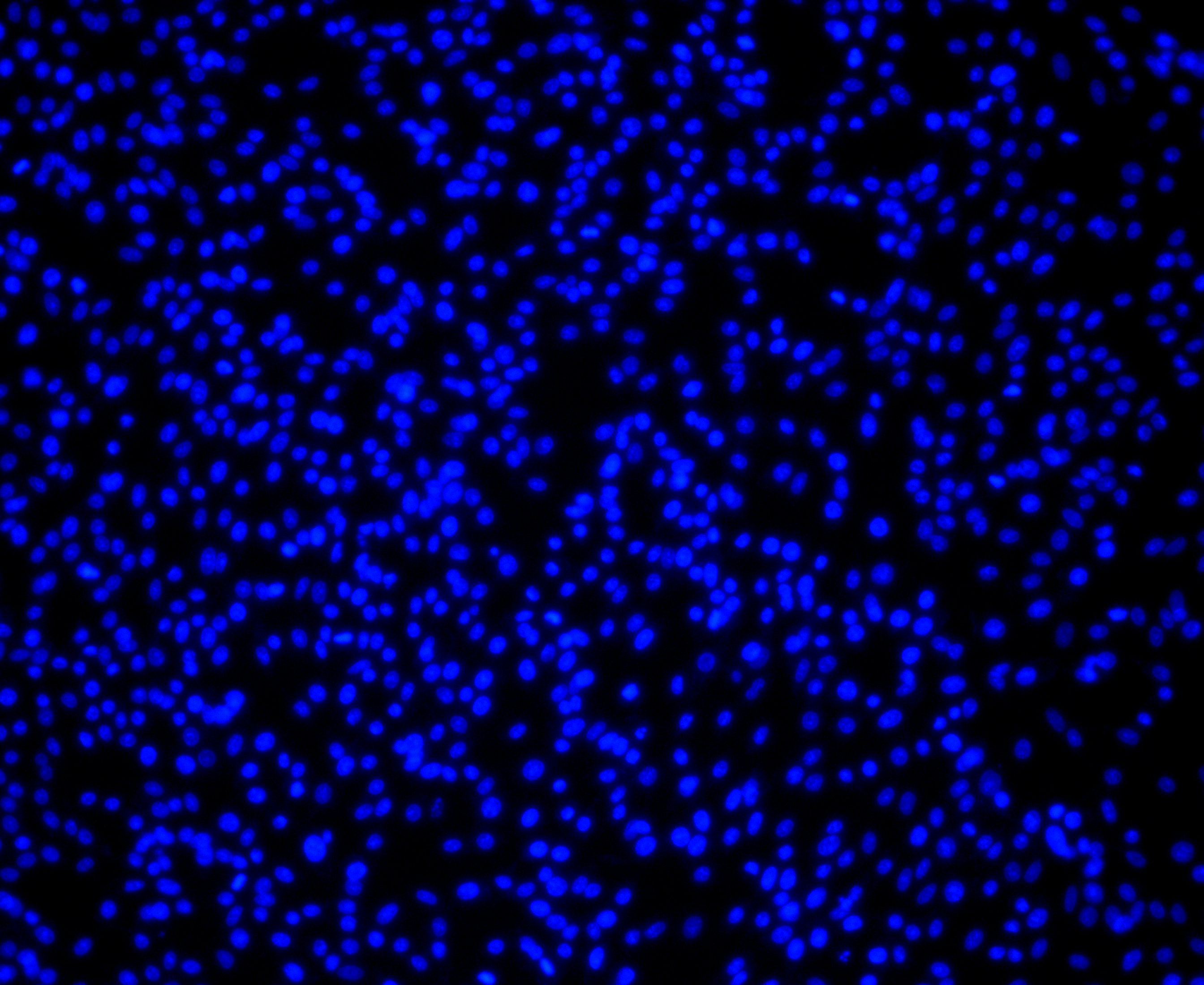

Supplement: Supplemental Material [file KBIE_A_2048775_SM5166.zip › Fig6A_LPS_Si_Lrg1_DAPI.tif]

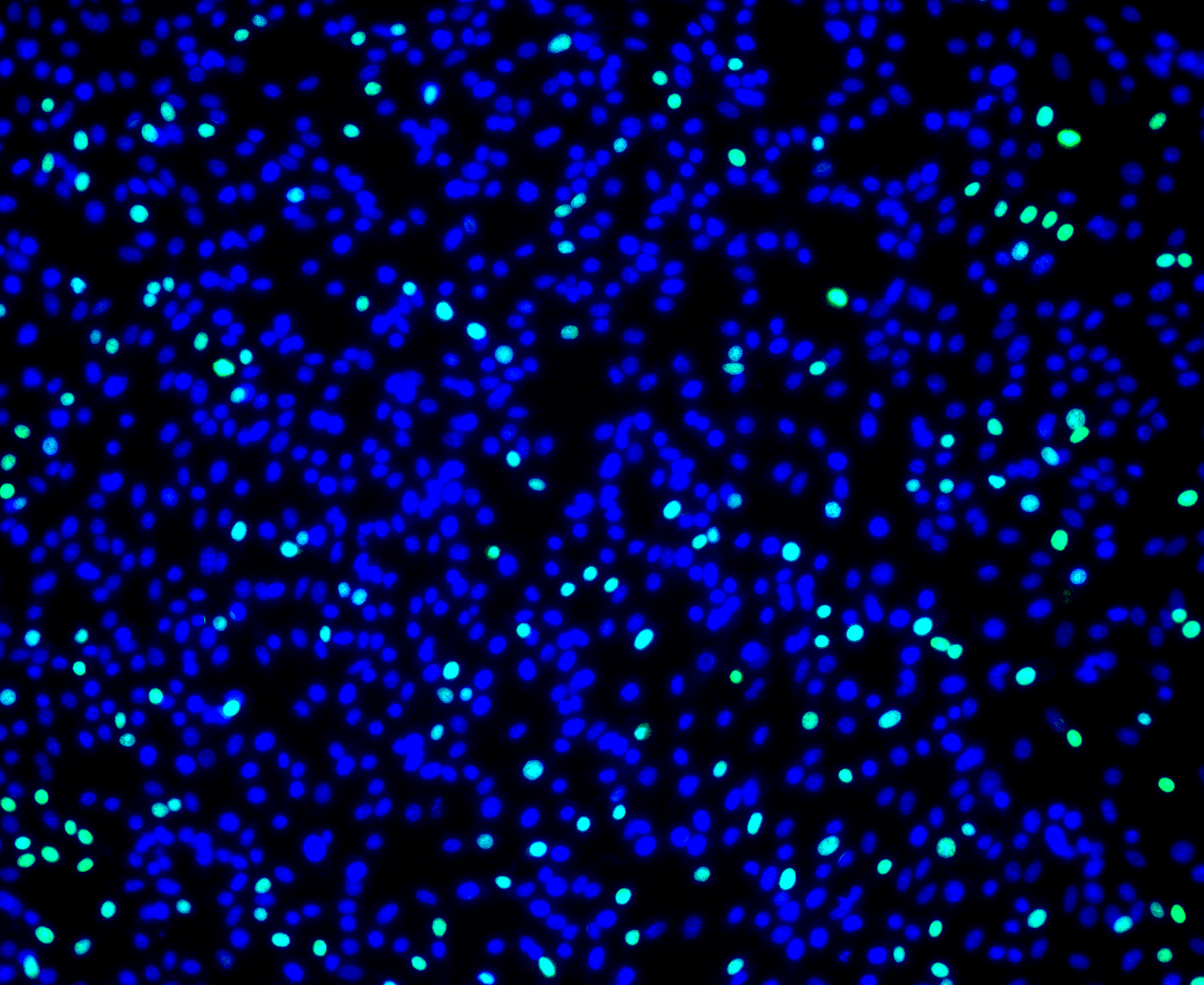

Supplement: Supplemental Material [file KBIE_A_2048775_SM5166.zip › Fig6A_LPS_Si_Lrg1_Merged.tif]

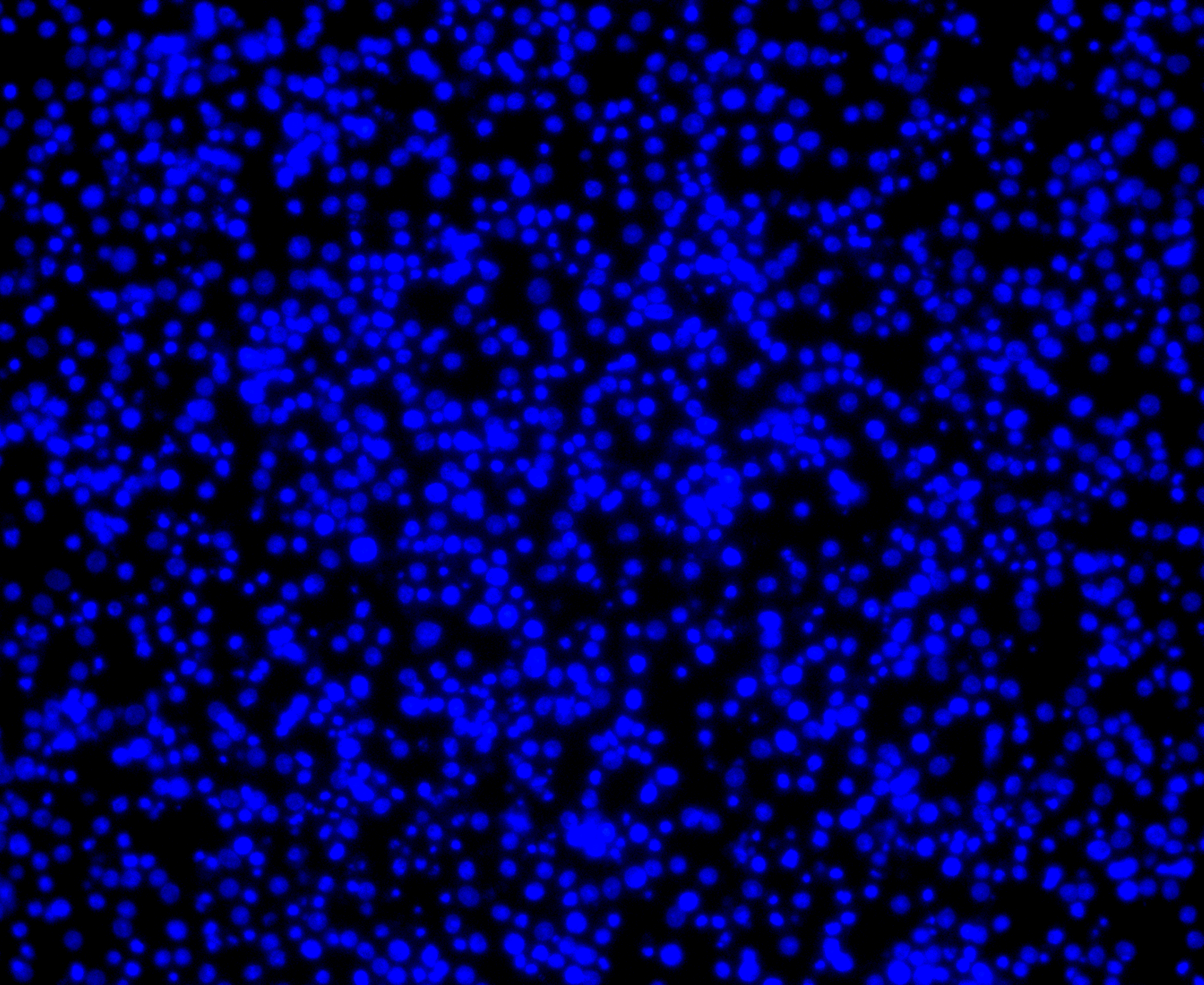

Supplement: Supplemental Material [file KBIE_A_2048775_SM5166.zip › Fig6A_LPS_Si_Lrg1_Oe_NC_DAPI.tif]

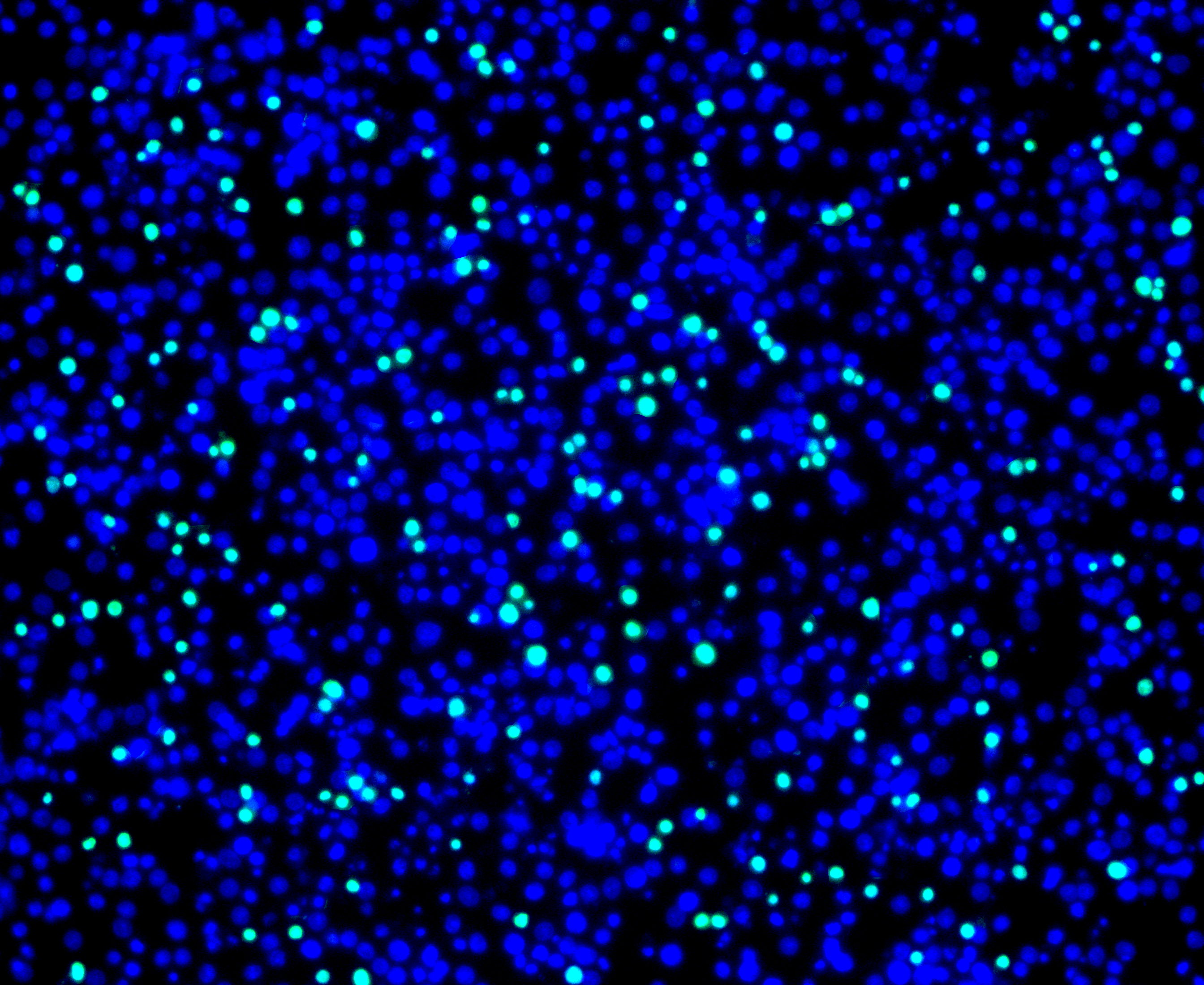

Supplement: Supplemental Material [file KBIE_A_2048775_SM5166.zip › Fig6A_LPS_Si_Lrg1_Oe_NC_Merged.tif]

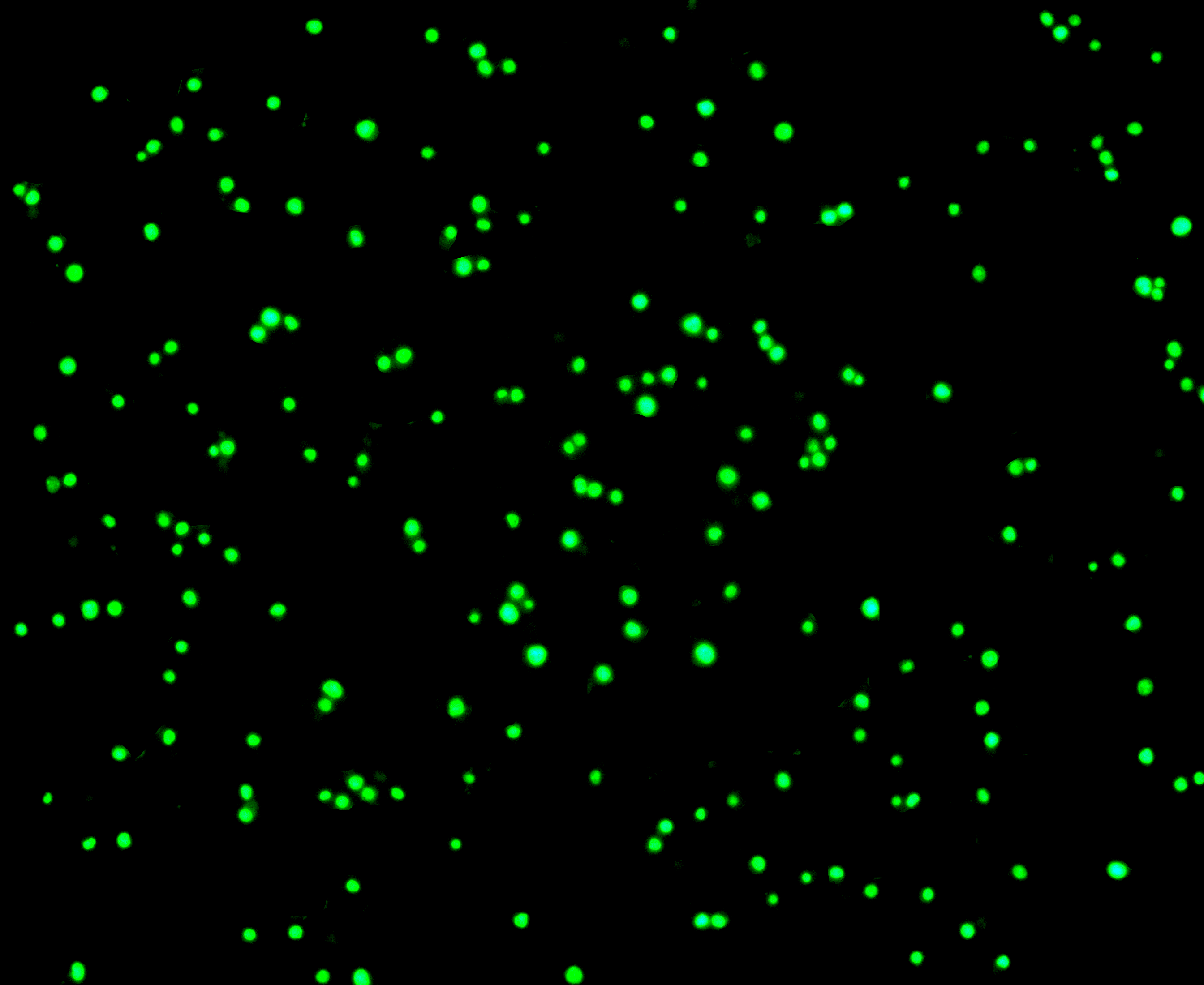

Supplement: Supplemental Material [file KBIE_A_2048775_SM5166.zip › Fig6A_LPS_Si_Lrg1_Oe_NC_TUNEL.tif]

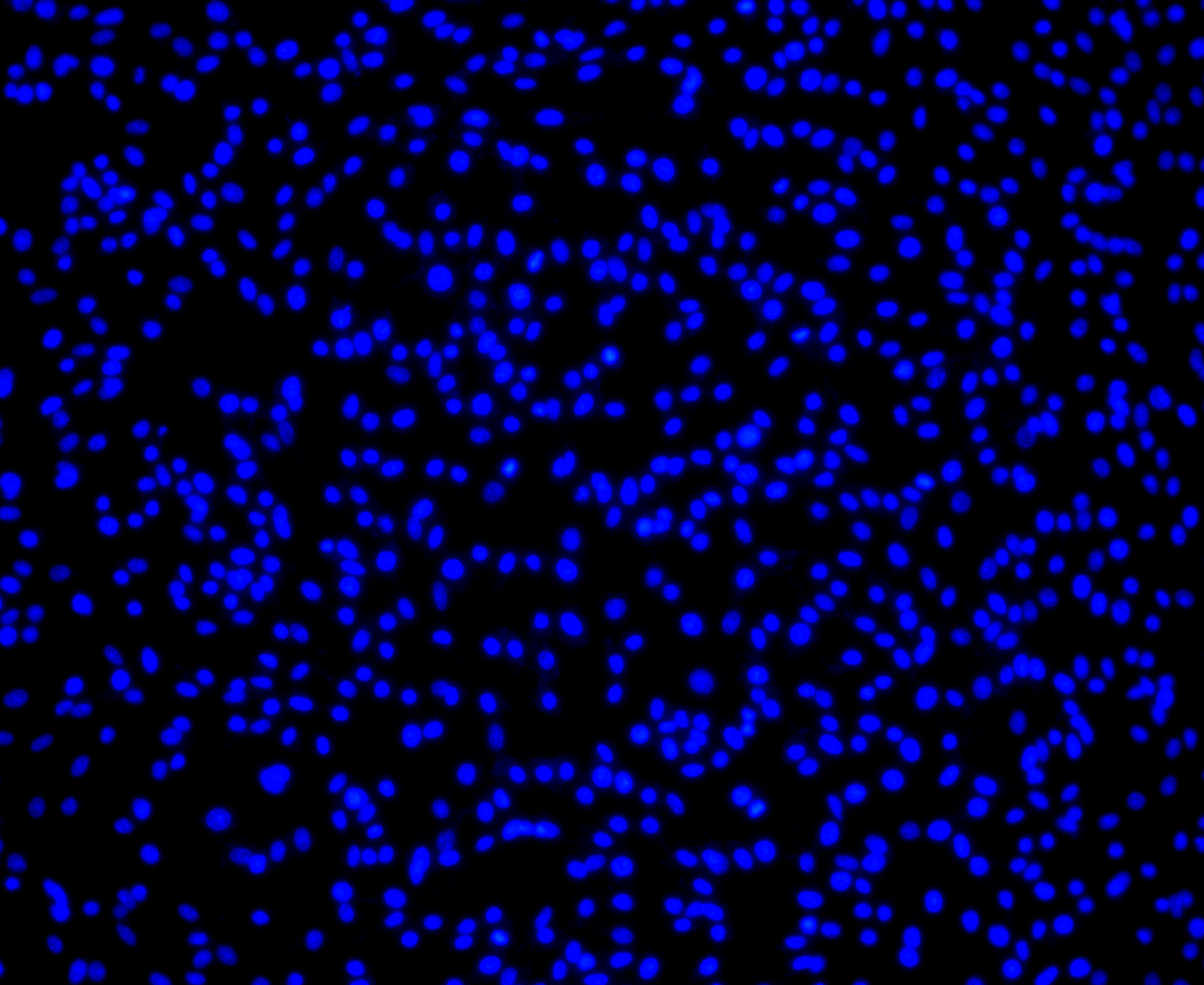

Supplement: Supplemental Material [file KBIE_A_2048775_SM5166.zip › Fig6A_LPS_Si_Lrg1_Oe_TGFb1_DAPI.tif]

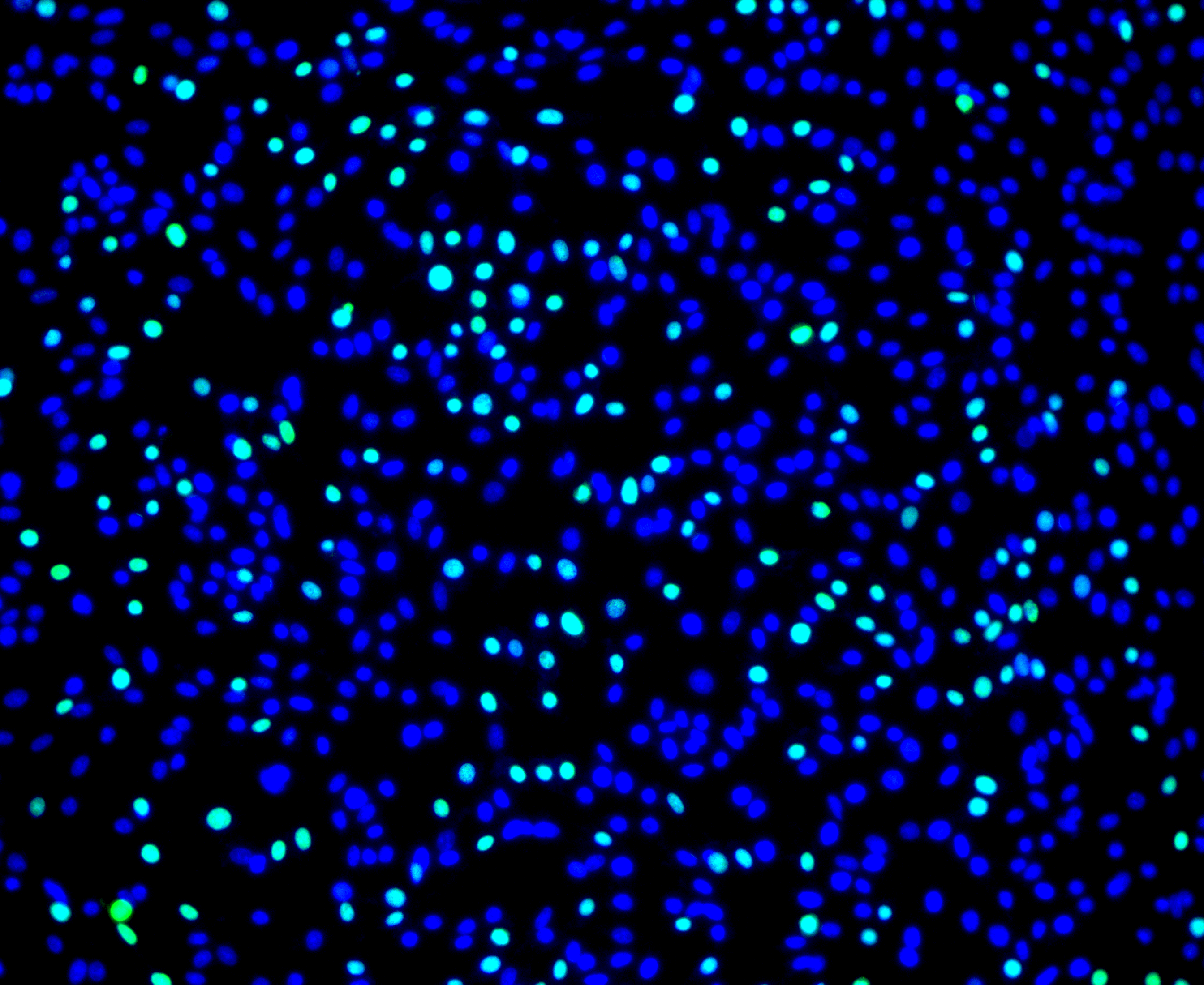

Supplement: Supplemental Material [file KBIE_A_2048775_SM5166.zip › Fig6A_LPS_Si_Lrg1_Oe_TGFb1_Merged.tif]

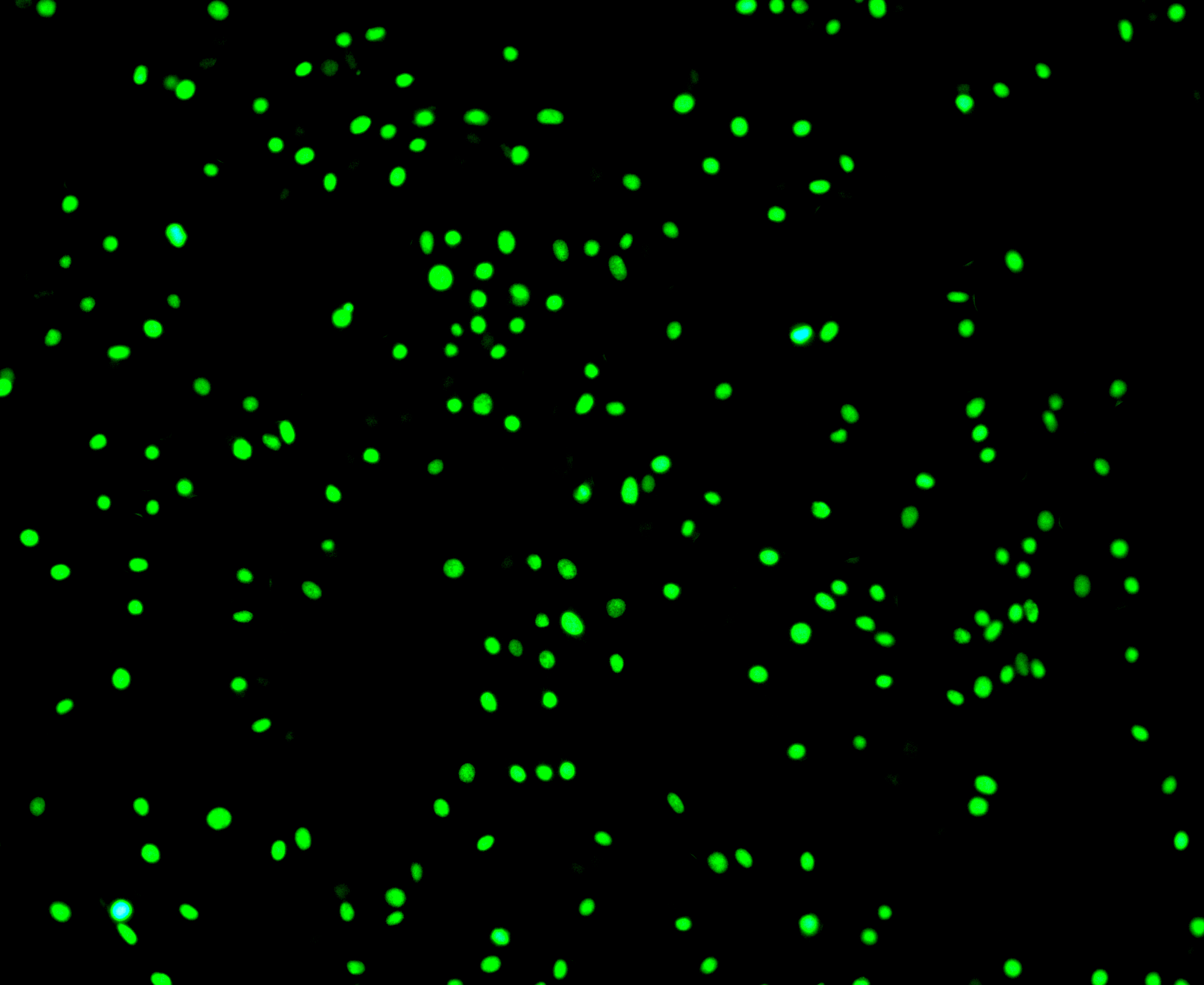

Supplement: Supplemental Material [file KBIE_A_2048775_SM5166.zip › Fig6A_LPS_Si_Lrg1_Oe_TGFb1_TUNEL.tif]

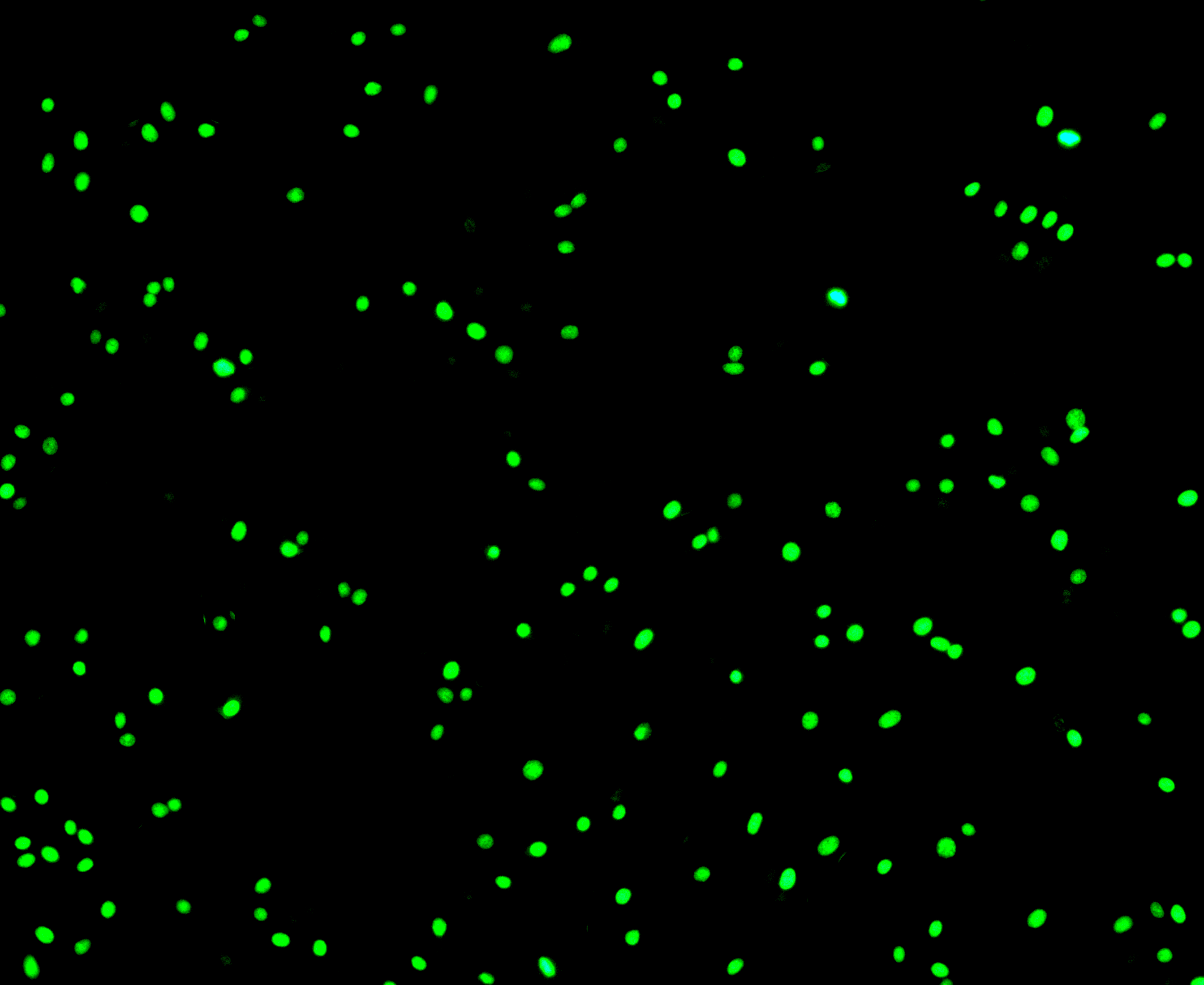

Supplement: Supplemental Material [file KBIE_A_2048775_SM5166.zip › Fig6A_LPS_Si_Lrg1_TUNEL.tif]

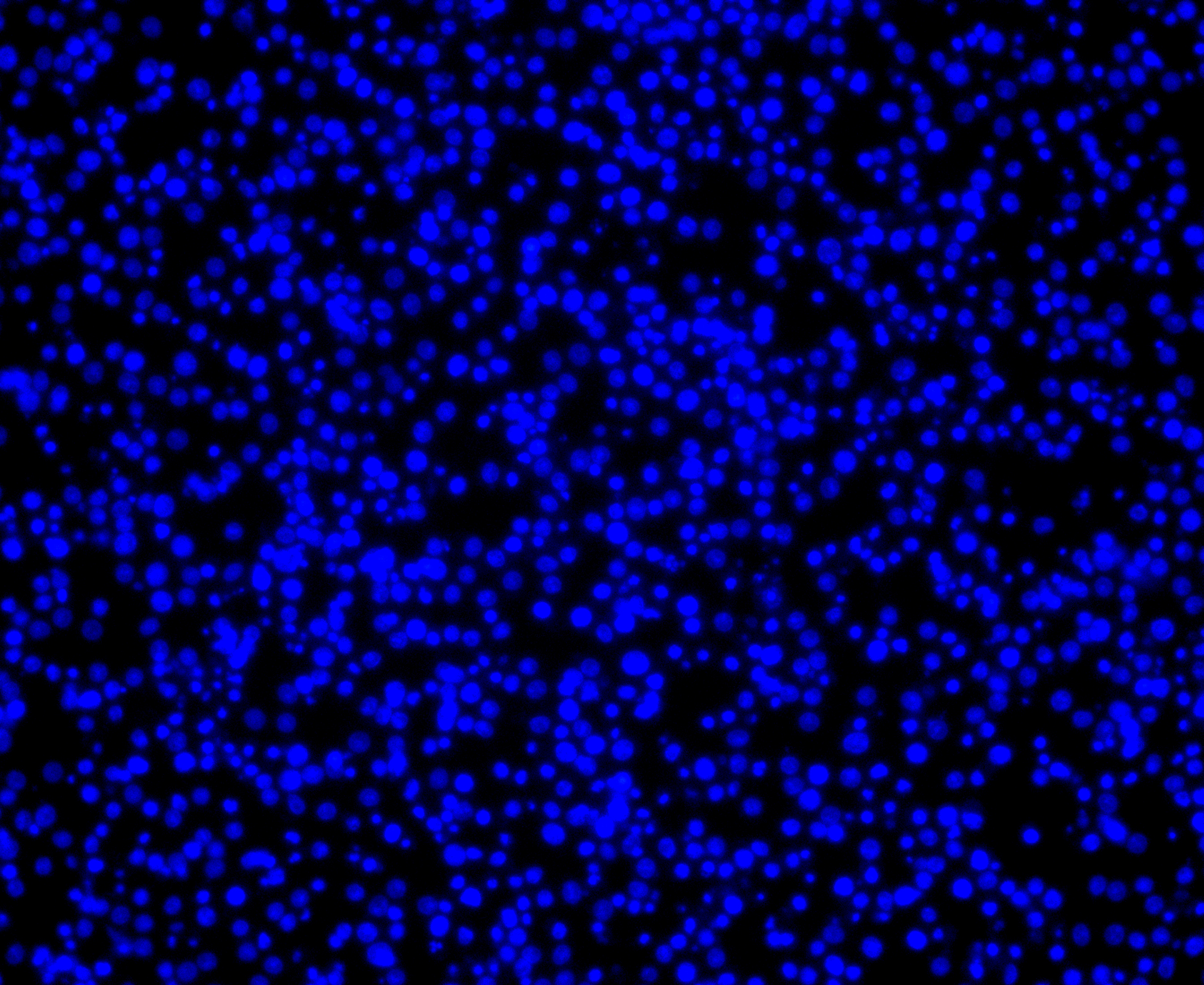

Supplement: Supplemental Material [file KBIE_A_2048775_SM5166.zip › Fig6A_LPS_Si_NC_DAPI.tif]

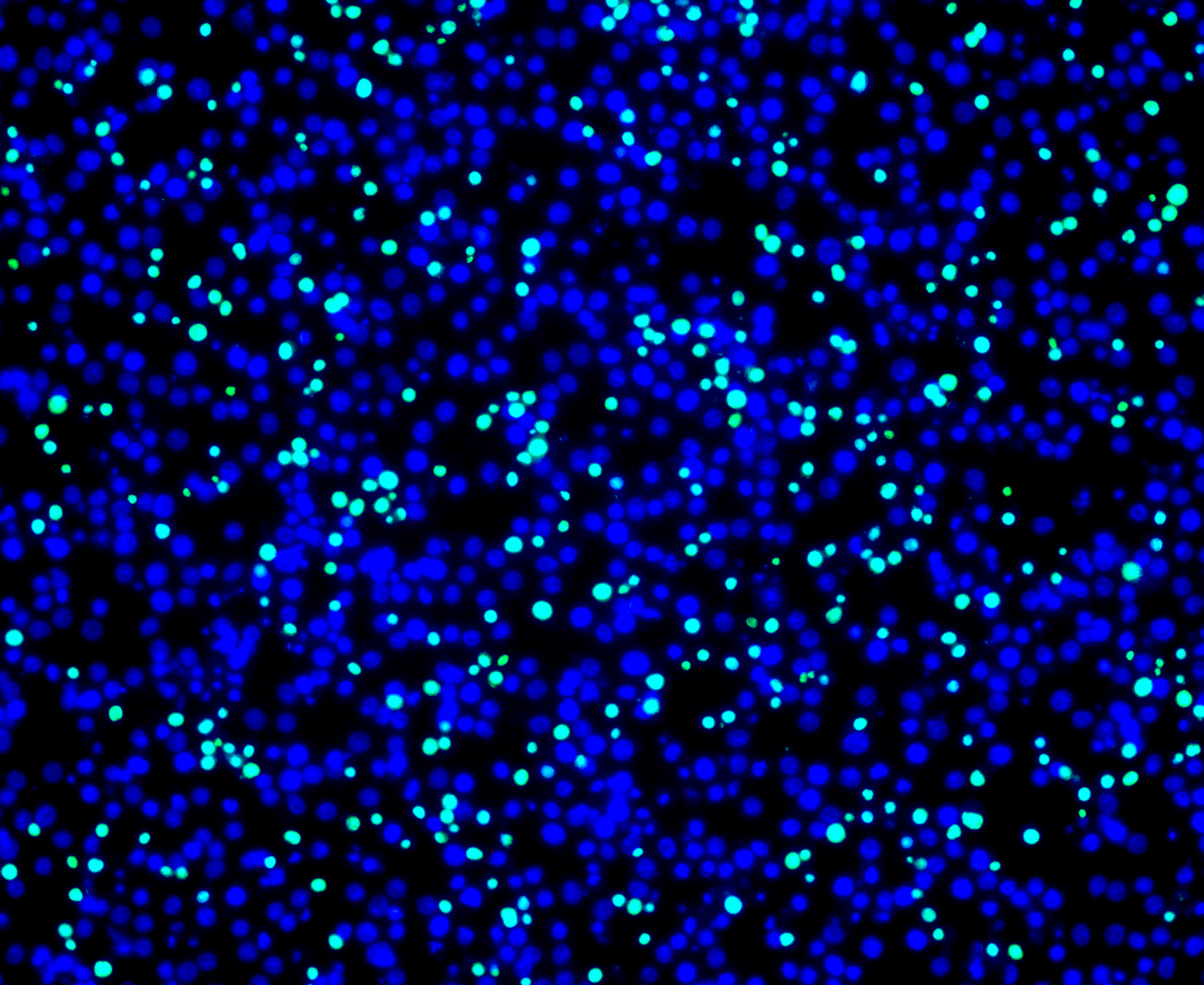

Supplement: Supplemental Material [file KBIE_A_2048775_SM5166.zip › Fig6A_LPS_Si_NC_Merged.tif]

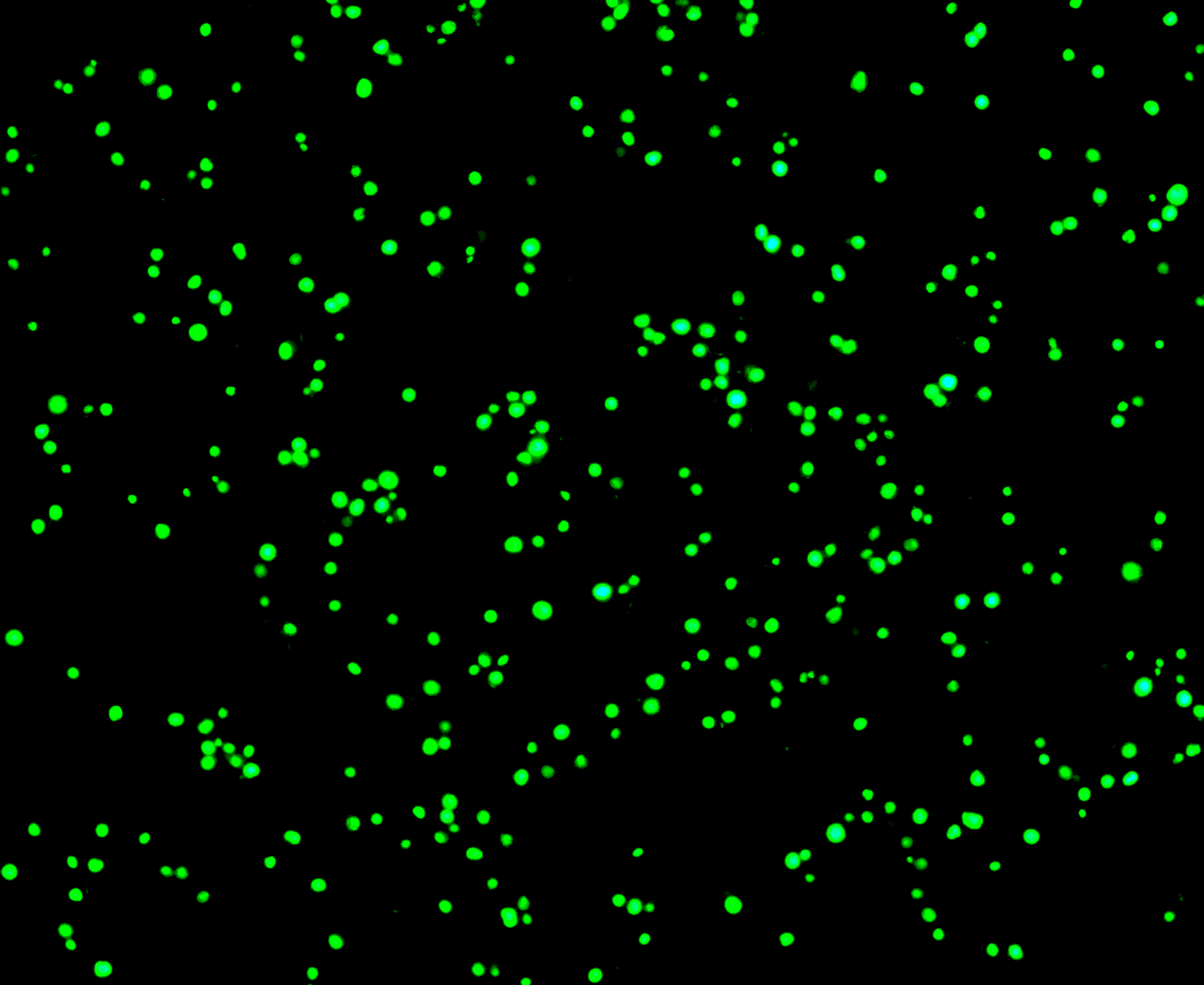

Supplement: Supplemental Material [file KBIE_A_2048775_SM5166.zip › Fig6A_LPS_Si_NC_TUNEL.tif]

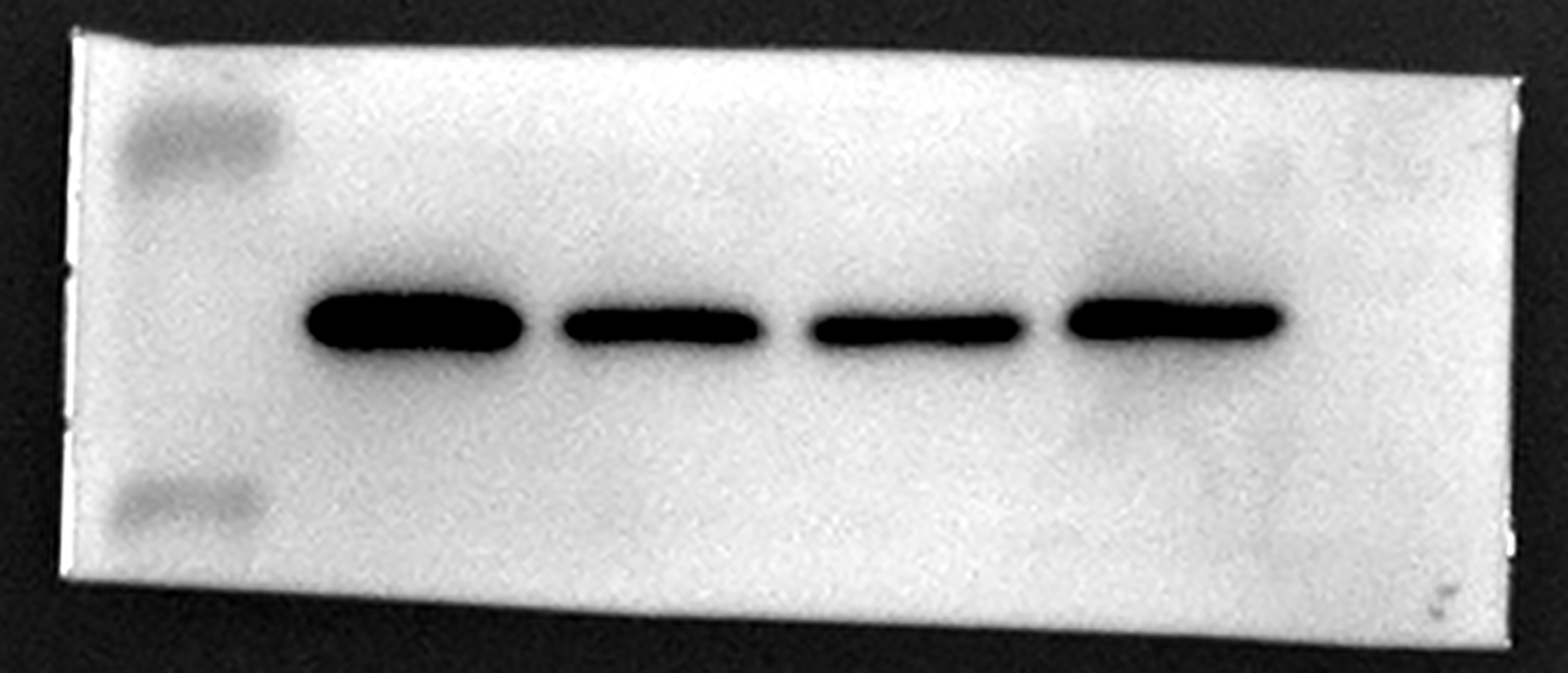

Supplement: Supplemental Material [file KBIE_A_2048775_SM5166.zip › Fig6B_Bax.tif]

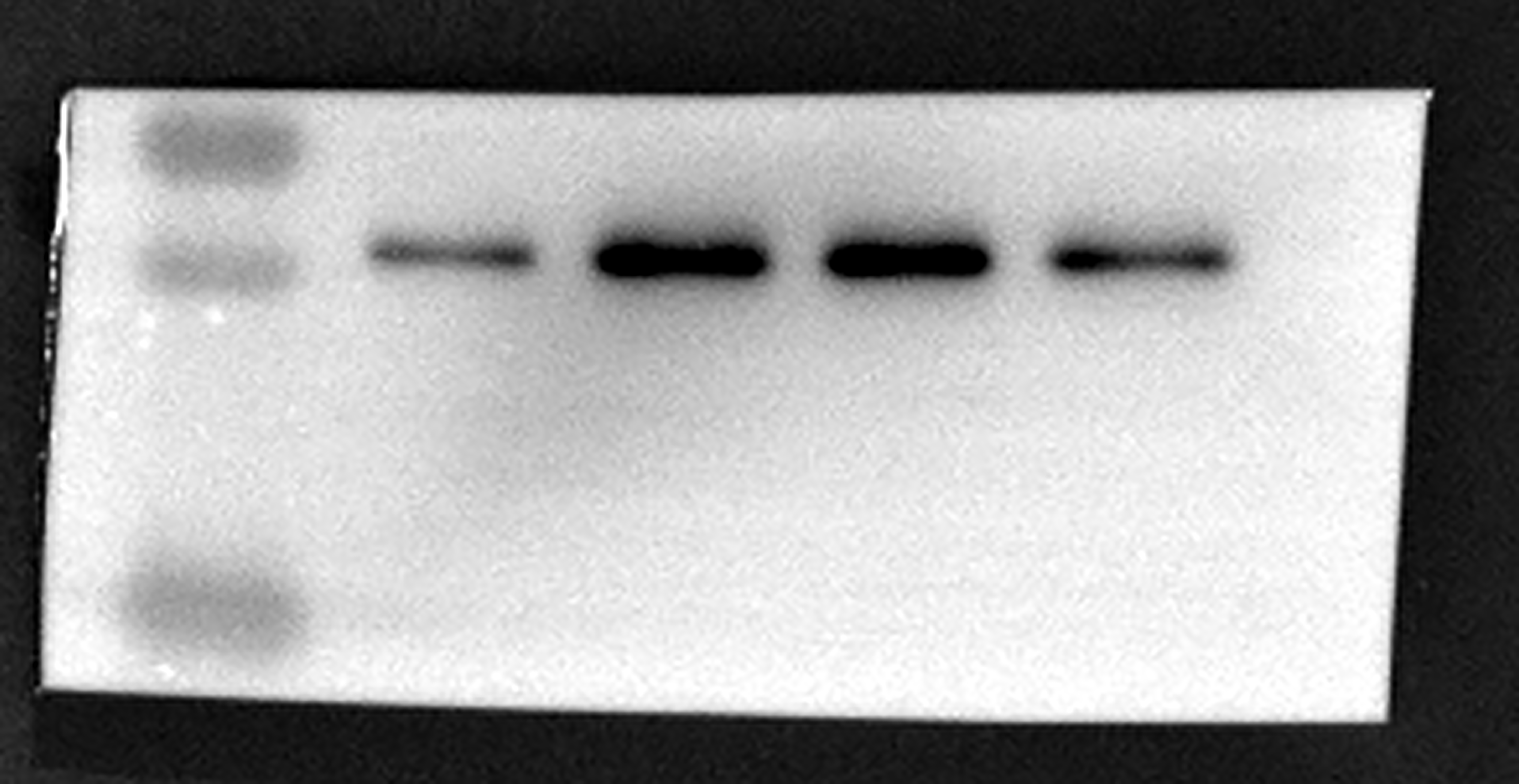

Supplement: Supplemental Material [file KBIE_A_2048775_SM5166.zip › Fig6B_Bcl_2.tif]

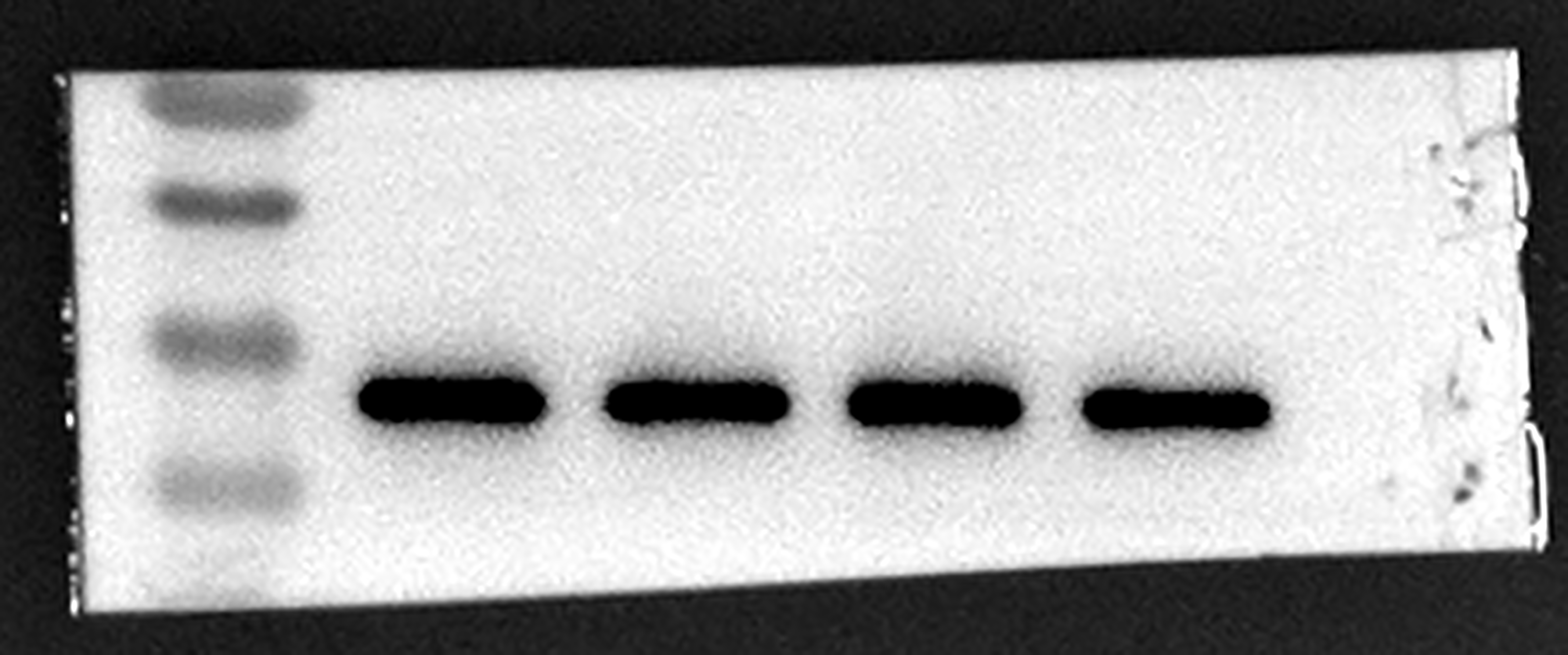

Supplement: Supplemental Material [file KBIE_A_2048775_SM5166.zip › Fig6B_caspase3.tif]
